# Ligand Arm Length Governs Geometry, Spin State, and Reactivity in Unsymmetrical β‑Diketiminato Nickel(II) and Nickel(I) Complexes

**DOI:** 10.1021/acs.inorgchem.6c03078

**Published:** 2026-08-28

**Authors:** Tzu-Hsien Yang, Si-Hong Chen, Yu-Ting Chu, Ming-Li Tsai, Hsing-Yin Chen, Sodio C. N. Hsu

**Affiliations:** † Department of Medicinal and Applied Chemistry, College of Life Sciences, 38023 Kaohsiung Medical University, Kaohsiung 80708, Taiwan; ‡ International PhD Program for Science, 34874 National Sun Yat-Sen University, Kaohsiung 80424, Taiwan; § Department of Chemistry, 34874 National Sun Yat-Sen University, Kaohsiung 80424, Taiwan; ∥ Department of Medical Research, Kaohsiung Medical University Hospital, Kaohsiung 80708, Taiwan

## Abstract

Unsymmetrical *N*-aryl-*N*′-alkylpyridyl β-diketiminate ligands (H**L1** and H**L2**) enable fine control over the coordination geometry, spin-state, and reactivity of nickel­(II) and nickel­(I) complexes through variation in pyridyl chelating arm length. Treatment of NiCl_2_(2,4-lutidine)_2_ with deprotonated ligands H**L1** and H**L2** in THF afforded the corresponding complexes **L1**Ni^II^Cl and **L2**Ni^II^Cl, respectively. X-ray crystallography reveals that **L1**Ni^II^Cl adopts a distorted square-planar geometry and is diamagnetic, whereas **L2**Ni^II^Cl exhibits the rare paramagnetic seesaw geometry due to the extended six-membered chelate ring. Reduction with KC_8_ yields the three-coordinate nickel­(I) complexes **L1**Ni^I^ (T-shaped) and **L2**Ni^I^ (Y-shaped), with rhombic EPR signals (g_1_ = 2.288–2.349) reflecting ligand-modulated spin density. Most notably, **L2**Ni^II^Cl undergoes unprecedented slow activation of the β-diketiminate backbone in acetonitrile, producing the high-spin five-coordinate adduct [**L2** ^MeCNH^NiCl]­[Cl], providing evidence for nitrile-induced ligand modification, a reactivity absent in the shorter-arm analogue **L1**Ni^II^Cl. In contrast, hydroxide coordination affords the mononuclear square-planar **L1**Ni^II^OH with a terminal OH group, whereas **L2**Ni^II^Cl forms the diamagnetic dimer **L2**Ni­(μ–OH)_2_Ni**L2** with bridging hydroxides, demonstrating ligand-dependent nuclearity control. These results highlight how chelate ring size and ligand flexibility serve as powerful levers for tuning electronic structure, coordination geometry, spin multiplicity, and ligand-centered reactivity.

## Graphical abstract

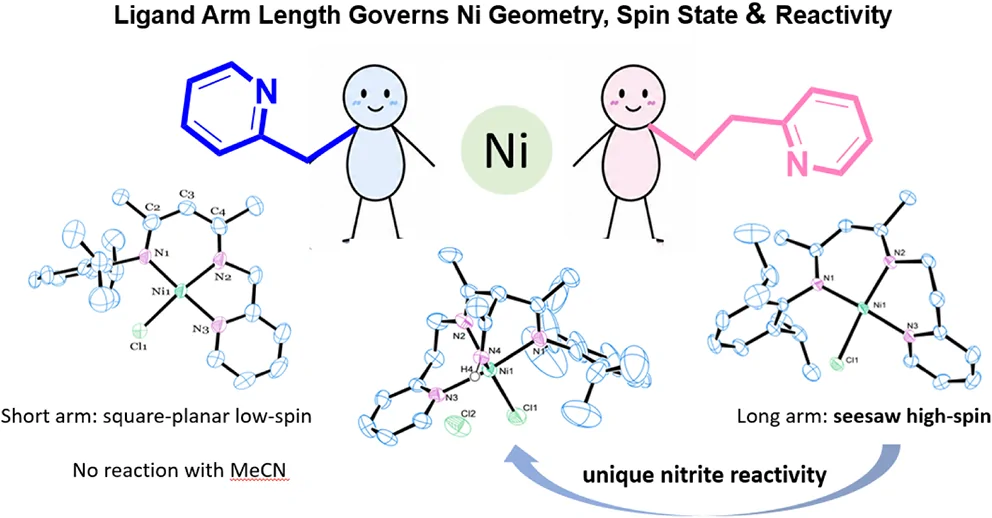

## Introduction

The geometry and spin-state of transition metal complexes are profoundly influenced by ligand design, which in turn governs their electronic structure and reactivity, a central theme in coordination chemistry.^1–10^ Ligands govern the spatial arrangement around the metal center, with bulkier ligands often inducing distortions in the coordination geometry.^11–22^ In contrast, multidentate ligands, featuring multiple binding sites, frequently form chelate rings, further influencing the overall geometry. Regarding spin-state, ligands modulate the electronic environment of the metal ion, altering the energy levels of its d-orbitals. Therefore, ligands serve as critical regulators of both geometry and spin-state, significantly impacting the structural and functional characteristics of transition metal complexes. Understanding and manipulating these ligand effects enables chemists to design and tailor metal complexes with precise properties for diverse applications in bioinspired coordination chemistry, materials science, and bioinorganic systems.^23–31^

Nickel complexes supported by β-diketiminate (nacnac or BDI) ligands have proven versatile platforms for exploring such ligand control, owing to the ligand’s tunable steric and electronic properties.^19,20,32–35^ While symmetric β-diketiminates have yielded diverse nickel species, including low-coordinate Ni­(I) complexes capable of activating H_2_, N_2_, and other small molecules, unsymmetrical variants remain underexplored for nickel.^21,22,36–40^ Our prior work on unsymmetrical *N*-aryl-*N′*-alkylpyridyl β-diketiminate ligands (H**L1** and H**L2**; Chart [Fig chart1]) demonstrated that extending the pyridyl chelating arm dramatically alters reactivity and geometry in copper complexes. For example, shorter-arm **L1** supports distorted square-planar Cu­(II) (τ_4_ = 0.16), while longer-arm **L2** favors distorted seesaw geometry (τ_4_ = 0.46).^28,30^ The pyridyl arm length also governs small-molecule binding (e.g., CO to Cu­(I)) and ligand degradation pathways in Cu­(I)-O_2_ chemistry.^28,29^ More recently, we demonstrated that an unsymmetrical *N*-aryl-*N′*-alkylpyridyl β-diketiminate ligand can stabilize a rare terminal nickel–oxygen species, which was crystallographically characterized as a resonance hybrid of Ni^III^=O and Ni^II^–O^•^ states. The flexible pyridyl arm was proposed to function as a hemilabile donor that enables geometric adaptation and stabilization of reactive nickel–oxygen intermediates. This finding further highlights the unique potential of unsymmetrical β-diketiminate frameworks for tuning the electronic structure and reactivity of nickel complexes.^41^

**1 chart1:**
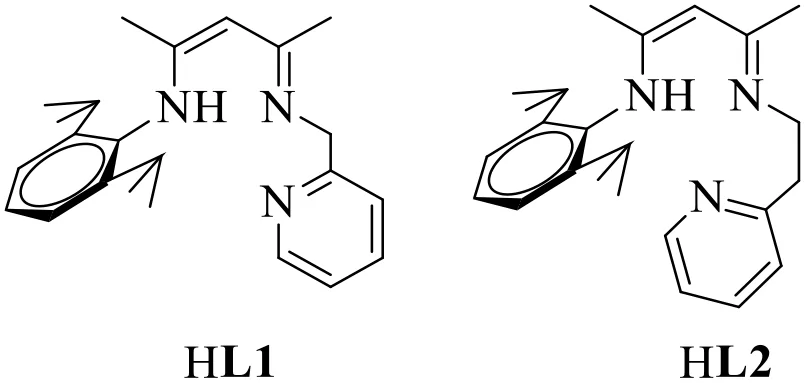
Unsymmetrical *N*-Aryl-*N*′-Alkyl β-Diketiminate Ligand Framework

Inspired by these findings, we hypothesized that analogous arm-length modulation in nickel β-diketiminates could enable precise tuning of coordination geometry (e.g., square-planar vs rare paramagnetic seesaw in Ni­(II)),^42–45^ spin state (diamagnetic vs high-spin), and reactivity, including access to distinct Ni­(I) species and unprecedented ligand backbone transformations. Herein, we report a systematic investigation of nickel­(II) and nickel­(I) complexes supported by H**L1** and H**L2**. The results reveal ligand-driven geometric isomerism (square-planar to seesaw in Ni­(II); T-shaped to Y-shaped in Ni­(I)), spin-state switching, and distinctive reactivity patterns, such as nitrile-induced β-C–C bond activation and ligand cleavage in the longer-arm system, as well as controlled nuclearity in hydroxide derivatives. These findings underscore the power of unsymmetrical β-diketiminate design in modulating nickel electronic structure and function.

## Results and Discussion

### Synthesis and Characterization of Nickel­(II) Complexes

The unsymmetrical β-diketiminato ligands H**L1** and H**L2** were deprotonated and coordinated to nickel using NiCl_2_(2,4-lutidine)_2_ in THF. For H**L1**, addition of KO^*t*^Bu afforded brown **L1**Ni^II^Cl in 79% yield (Scheme 1). In contrast, the longer pyridyl arm in H**L2** required the milder base NaOMe to yield reddish-black **L2**Ni^II^Cl in 67% yield. Note that **L1**Ni^II^Cl has been independently reported by Sen and co-workers^31^ using a different route, while the synthesis described here follows the same route as that previously reported by our laboratory.^41^ The ^1^H NMR spectroscopy confirmed coordination; the amine NH signal of H**L1** (11.32 ppm) disappeared, pyridine resonances sharpened and split, and the backbone methine shifted downfield from 4.77 to 4.91 ppm (Figure S1). **L1**Ni^II^Cl is diamagnetic, consistent with square-planar d^8^ Ni­(II). **L2**Ni^II^Cl, however, is paramagnetic (Figure S3), a rare feature for four-coordinate Ni­(II) complexes. The magnetic moment, determined by the Evans method in benzene (2.84 μ_B_, ∼2.01 unpaired electrons at 25 °C) and corroborated by SQUID (μ_*eff*_ = 2.571 μ_B_, ∼1.76 unpaired electrons; Figures S7 and S9), indicates two unpaired electrons (spin-only value for high-spin d^8^). This paramagnetism arises from distortion to seesaw geometry due to the extended chelating arm (vide infra). ESI-MS analysis identified a [**L1**Ni]^+^ peak at *m*/*z* 406.15 for **L1**Ni^II^Cl (Figure S20), while **L2**Ni^II^Cl showed a [**L2**NiCl + H]^+^ peak at *m*/*z* 456.13, validated by high-resolution mass spectrometry (HR-MS) at *m*/*z* = 456.1711, aligning with the simulated isotopic pattern (Figure S21).

**Scheme 1:**
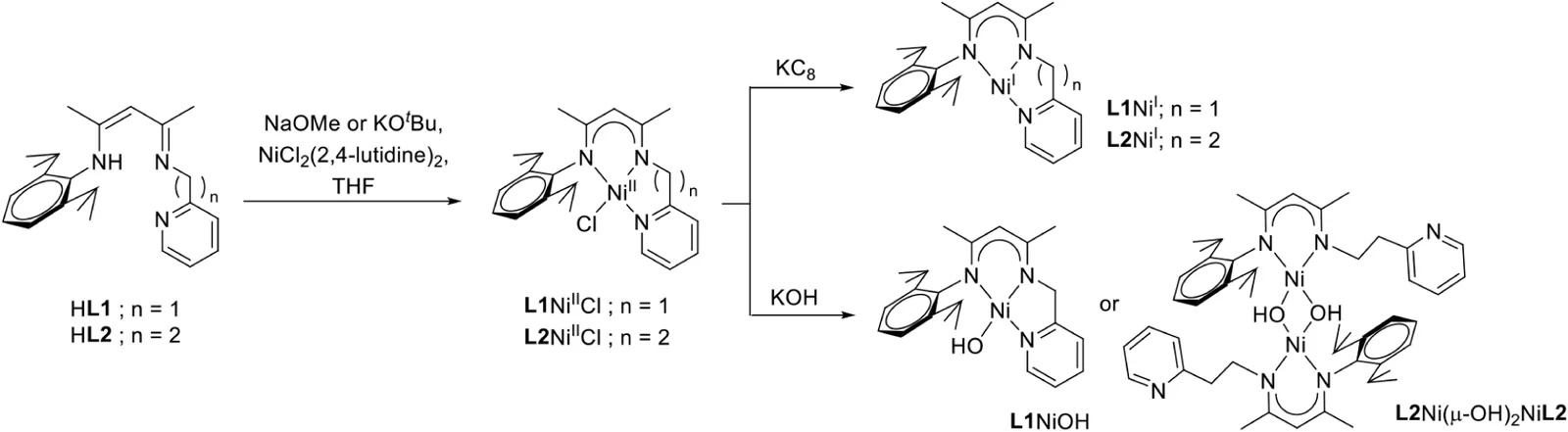
Synthetic Routes to the Nickel Complexes Described in This Work

### Structural Characterization of Nickel­(II) Complexes

Single crystals suitable for X-ray diffraction analysis of the nickel­(II) complexes were grown from MeCN (**L1**Ni^II^Cl)^41^ or THF (**L2**Ni^II^Cl) at −20 °C and revealed striking ligand-dependent geometries (Figure 1). **L1**Ni^II^Cl adopts distorted square-planar coordination (τ_4_ = 0.102), enforced by a rigid five-membered pyridyl-Ni chelate.^41^ **L2**Ni^II^Cl displays distorted seesaw geometry (τ_4_ = 0.299), driven by the more flexible six-membered chelate from the ethyl linker. Seesaw geometries are uncommon in four-coordinate Ni­(II), typically requiring specialized ligands (e.g., NNN pincer, PNP pincer, NHC ligand).^42–45^ The pyridyl arm length thus dictates geometry; shorter arms favor planarity, longer arms induce distortion. Out-of-plane displacements further highlight the effect; Ni in **L1**Ni^II^Cl lies 0.148 Å above the NCCCN plane (Cl 0.385 Å above NNNNi), while in **L2**Ni^II^Cl, Ni is 0.430 Å below and Cl 1.242 Å above. Notably, the Ni–N2 elongates from 1.840(3) Å (**L1**Ni^II^Cl) to 1.8780(19) Å (**L2**Ni^II^Cl), reflecting the structural impact of the extended chelating arm (Table 1). The crystal structure of **L1**Ni^II^Cl has been reported previously;^41^ however, it is included here for direct comparison with the newly synthesized **L2**Ni^II^Cl to elucidate the influence of pyridyl arm length on the coordination geometry and electronic structure of the nickel­(II) center.

**Figure 1:**
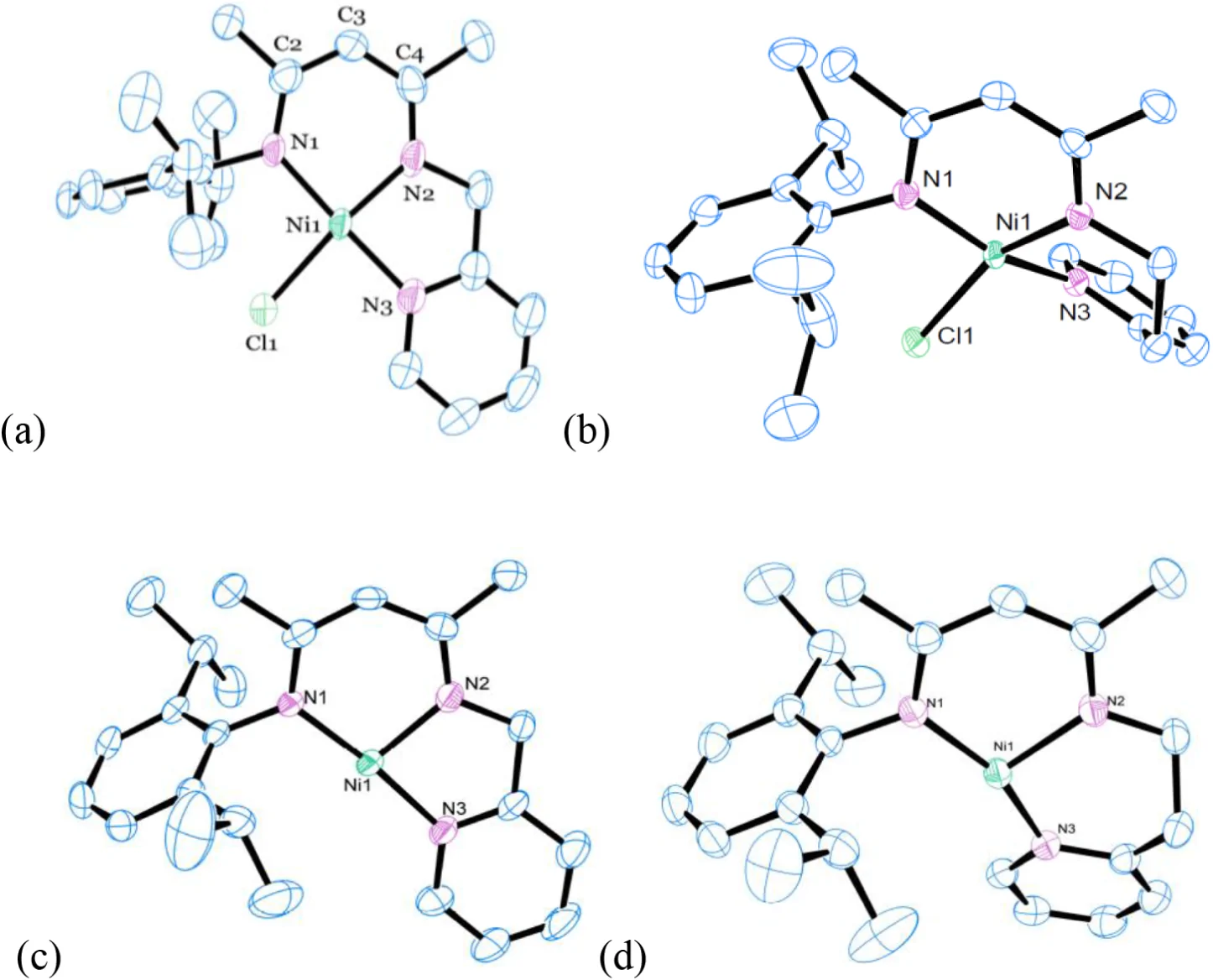
ORTEP drawings of (a) **L1**Ni^II^Cl, (b) **L2**Ni^II^Cl, (c) **L1**Ni^I^, and (d) **L2**Ni^I^. Thermal ellipsoids are drawn at the 50% probability level; hydrogen atoms are omitted for clarity. The structures of **L1**Ni^II^Cl and **L1**Ni^I^ were reported previously.^41^

**Table 1:** Selected Bond Length (Å) and Angles (Degrees) for **L1**Ni^II^Cl, **L2**Ni^II^Cl, **L1**Ni^I^, and **L2**Ni^I^

	**L1**Ni^II^Cl	**L2**Ni^II^Cl	**L1**Ni^I^	**L2**Ni^I^
N(1)–Ni(1)	1.879(3)	1.8948(18)	1.863(3)	1.8665(15)
N(2)–Ni(1)	1.848(3)	1.8780(19)	1.898(3)	1.9210(15)
N(3)–Ni(1)	1.907(3)	1.9113(19)	1.897(3)	1.9083(15)
Cl(1)–Ni(1)	2.2294(10)	2.2137(6)		
N(1)–Ni(1)–N(2)	89.63(12)	94.24(8)	98.98(12)	98.63(6)
N(2)–Ni(1)–N(3)	87.92(12)	90.41(8)	85.73(13)	101.23(7)
N(1)–Ni(1)–N(3)	175.13(13)	159.91(8)	173.64(12)	159.37(6)
N(2)–Ni(1)–Cl(1)	170.49(9)	157.96(6)		

### Preparation of Nickel­(I) Complexes

In biological systems, nickel-containing enzymes rely on nickel­(I) as the active catalytic center, underscoring its significance in metalloenzyme function.^20,46,47^ Recent renewed interest among chemists in Ni­(I) complexes has been tempered by the scarcity of studies that successfully isolate and characterize these species.^38,48,49^ To enhance the reactivity and utility of nickel-based complexes, we reduced β-diketiminato nickel­(II) complexes **L1**Ni^II^Cl and **L2**Ni^II^Cl to their nickel­(I) counterparts using KC_8_, resulting in the paramagnetic species **L1**Ni^I^ and **L2**Ni^I^ (Scheme 1). Note that **L1**Ni^I^ has been independently reported using an alternative synthetic approach,^50^ while the synthesis described here follows the same route as that previously reported by our laboratory.^41^

The EPR spectrum of **L2**Ni^I^ recorded in toluene exhibits a rhombic signal with g values of g_1_ = 2.349, g_2_ = 2.141, and g_3_ = 2.042 (Figure S11). These g values deviate from the free-electron g value (∼2.0023), indicating a significant contribution of the nickel d orbitals to the singly occupied molecular orbital (SOMO), consistent with a Ni­(I) (d^9^) electronic configuration. In addition, **L2**Ni^I^ exhibits resolved ^14^N superhyperfine splitting that is very similar to that observed for **L1**Ni^I^. In our previous work, the rhombic EPR signal of **L1**Ni^I^ was assigned to superhyperfine coupling with one ligand ^14^N nucleus (A_N_ = 17 G).^41^ **L2**Ni^I^ displays a very similar splitting pattern with a slightly smaller coupling constant (A_N_ = 14 G), supporting a comparable superhyperfine interaction. This observation is consistent with the EPR features reported for analogous three-coordinate nickel­(I) complexes.^49,51,52^ The subtle difference in the g_1_ values of **L1**Ni^I^ and **L2**Ni^I^ (2.288 vs 2.349) suggests ligand-dependent modulation of spin density and orbital contributions, arising from differences in chelate ring size and the steric/electronic effects of the pyridyl arm.

### Structural Characterization of Nickel­(I) Complexes

Single crystals of **L1**Ni^I^ and **L2**Ni^I^ suitable for X-ray diffraction analysis were obtained by slow cooling of saturated pentane solutions to −20 °C. The structures reveal distinctly different three-coordinate geometries dictated by the pyridyl arm length (Figure 1). **L1**Ni^I^ adopts a T-shaped coordination geometry,^41^ whereas **L2**Ni^I^ displays a Y-shaped arrangement, both of which are typical of low-coordinate d^9^ Ni­(I) species but rarely differentiated within the same ligand family.

Selected bond lengths and angles are summarized in Table 1. The central Ni–N2 bond (the nitrogen of the β-diketiminate backbone) is noticeably longer in **L2**Ni^I^ [1.9210(15) Å] than in **L1**Ni^I^ [1.898(3) Å], consistent with reduced π-donation or increased steric pressure from the extended ethyl linker. Out-of-plane displacement of the nickel center from the NCCCN mean plane of the β-diketiminate ligand is also significantly greater in **L2**Ni^I^ (0.099 Å) than in **L1**Ni^I^ (0.018 Å), reflecting the increased conformational flexibility and steric demand imposed by the six-membered chelate ring versus the more rigid five-membered ring in **L1**. These structural differences highlight the critical role of ligand asymmetry and pyridyl arm length in modulating the coordination environment of nickel­(I) centers. While the crystal structure of **L1**Ni^I^ has been reported previously,^41^ its inclusion here provides a direct structural benchmark for **L2**Ni^I^ and underscores the profound influence of pyridyl arm length on the geometry of three-coordinate nickel­(I) species.

### Reactivity of L2Ni^II^Cl toward Acetonitrile

The longer-arm complex **L2**Ni^II^Cl displays remarkable and unprecedented reactivity in acetonitrile, resulting in activation of the β-diketiminate ligand backbone. Dissolution of **L2**Ni^II^Cl in MeCN at room temperature produces no immediate color change, but the solution slowly turns green over several days, signaling the gradual formation of new nickel-containing species. After ∼1 week, solvent removal and benzene washing afforded light-blue crystals of the ionic complex [**L2** ^MeCNH^NiCl]­[Cl] in 33% isolated yield (Scheme 2). The transformation is significantly accelerated by the addition of an external proton source. Introduction of HCl in diethyl ether to an acetonitrile solution of **L2**Ni^II^Cl induces a rapid color change and increases the isolated yield of [**L2** ^MeCNH^NiCl]­[Cl] to 46%. These observations imply protonation plays an important role in facilitating the nitrile activation and subsequent ligand modification.

**Scheme 2:**
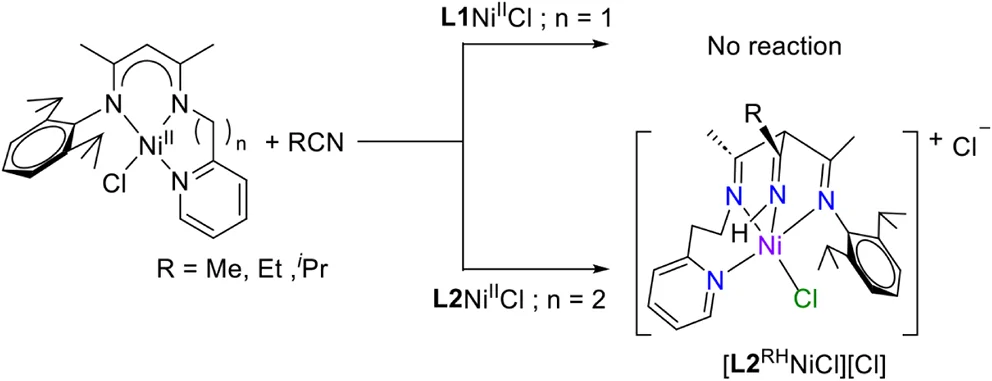
Nitrile-Induced Ligand Backbone Activation of L2Ni^II^Cl to Form Ionic Complexes [L2^RH^NiCl]­[Cl] (R = Me, Et, or ^*i*^Pr); No Reaction Is Observed for the Shorter-Arm Analogue L1Ni^II^Cl under Identical Conditions

ESI-MS analysis of the reaction mixture revealed a dominant ion peak at *m*/*z* = 497.1977, corresponding to the cationic species [**L2** ^MeCNH^NiCl]^+^, with an isotopic pattern matching the simulation (Figure S22). The reaction is general for alkyl nitriles, such as propionitrile and isobutyronitrile, which afford analogous adducts detected as [**L2** ^EtCNH^NiCl]^+^ (*m*/*z* = 511.08) and [**L2** ^iPrCNH^NiCl]^+^ (*m*/*z* = 525.08), respectively (Figure S23). These results indicate that coordination and activation extend beyond acetonitrile. The product [**L2** ^MeCNH^NiCl]­[Cl] is paramagnetic. ^1^H NMR spectra in CD_3_CN display broadened, strongly shifted resonances characteristic of a paramagnetic species (Figure S5). The effective magnetic moment, determined by the Evans method in acetonitrile at 25 °C, is 2.86 μ_B_, ∼2.03 unpaired electrons (Figure S8), consistent with a high-spin d^8^ Ni­(II) center possessing two unpaired electrons.

Control experiments with the shorter-arm analogue **L1**Ni^II^Cl reveal a stark contrast in behavior. ESI-MS analysis of **L1**Ni^II^Cl in acetonitrile (Figure S20) shows only the parent fragment ion [**L1**Ni]^+^ (*m*/*z* = 406.15), with no evidence for acetonitrile activation. Consistent with this observation, **L1**Ni^II^Cl remains stable in acetonitrile for at least 7 days at room temperature with no evidence of nitrile activation or ligand modification. Heating to 50 °C leads only to decomposition, and addition of HCl/Et_2_O produces a color change accompanied by unresolved NMR signals indicative of multiple decomposition products rather than a single well-defined adduct.

Supporting this interpretation, ESI-MS analysis of the reaction mixture reveals a new signal at *m*/*z* = 447.43, tentatively assigned to a MeCN-coordinated nickel species (Figure S24). To further support the assignment of the *m*/*z* = 447.43 species as a MeCN-coordinated nickel complex, an independent synthesis was performed. Treatment of **L1**Ni^II^Cl with AgCF_3_SO_3_ in acetonitrile afforded the cationic complex [**L1**Ni­(MeCN)]^+^[CF_3_SO_3_]^−^. ESI-MS analysis of this independently prepared sample shows a signal at *m*/*z* = 447.39 with an isotopic pattern consistent with simulation (Figure S25), in good agreement with the species observed in the reaction mixture. Single-crystal X-ray diffraction analysis further confirms the coordination of acetonitrile to the nickel center in [**L1**Ni­(MeCN)]^+^ (Figure 2a). The molecular structure reveals a four-coordinate nickel center with MeCN bound through the nitrile nitrogen, providing direct structural evidence for metal-bound acetonitrile in this system. In addition, a related acetonitrile-coordinated nickel complex has been reported by the Sen group, where an analogous structure was obtained using AgSbF_6_, differing only in the counteranion.^31^

**Figure 2:**
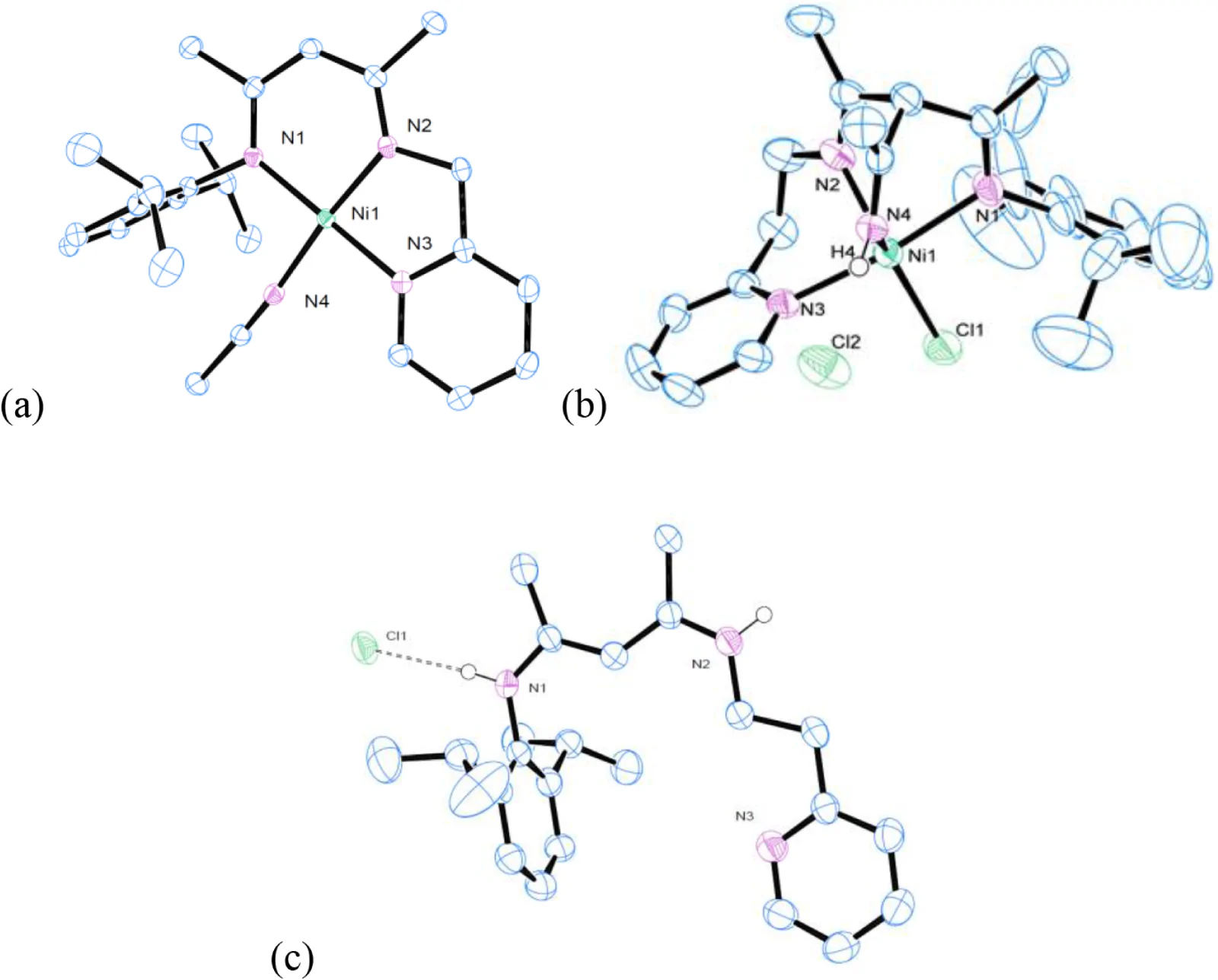
ORTEP drawings of complexes (a) the cationic complex [**L1**Ni­(MeCN)]^+^, (b) the ionic complex [**L2** ^MeCNH^NiCl]­[Cl], and (c) nickel-free ligand-derived byproduct. Thermal ellipsoids are drawn at the 50% probability level; hydrogen atoms are omitted for clarity.

However, the absence of an isolable product suggests that such coordination does not lead to productive nitrile activation, but instead results in unselective decomposition pathways. These results demonstrate that extension of the pyridyl chelating arm is essential for enabling this unusual nitrile-induced ligand backbone activation pathway in nickel β-diketiminate chemistry.

### Structural Characterization of Acetonitrile-Derived Products

Single-crystal X-ray diffraction analysis of the isolated light-blue product confirms its formulation as the ionic complex [**L2** ^MeCNH^NiCl]­[Cl] (Figure 2b). The nickel center adopts a distorted trigonal bipyramidal geometry (τ_5_ = 0.595), coordinated by three nitrogen atoms from the modified β-diketiminate ligand, one terminal chloride, and one nitrogen atom originating from the acetonitrile fragment. Key bond lengths include Ni–Cl = 2.2563(16) Å and Ni–N­(MeCN) = 2.003(4) Å. The equatorial angles N(2)–Ni(1)–Cl(1) = 137.51(13)° and N(2)–Ni(1)–N(3) = 87.26(17)° are consistent with significant distortion from ideal trigonal bipyramidal symmetry (Table S4). A weak intermolecular hydrogen bond is present between the protonated nitrile nitrogen H(4) and the chloride counteranion Cl(2) [H(4)···Cl(2) = 2.308 Å, N(4)···Cl(2) = 3.186 Å], stabilizing the ionic lattice.

A nickel-free ligand-derived byproduct was also structurally characterized from the same crystallization batch (Figure 2c). This species is a protonated, fragmented organic ligand derived from the dissociation and rearrangement of the original β-diketiminate framework. The isolation and crystallographic characterization of these two distinct species, [**L2** ^MeCNH^NiCl]­[Cl] and the nickel-free ligand fragment, provide direct structural evidence for the susceptibility of the longer-arm β-diketiminate ligand to nitrile-induced chemical transformation. The pronounced reactivity of **L2**Ni^II^Cl under these conditions, absent in the shorter-arm analogue **L1**Ni^II^Cl, underscores the critical influence of chelate ring size and ligand flexibility on enabling this unusual backbone activation pathway.

### Computational Investigation of the Acetonitrile Reactivity of L1Ni^II^Cl and L2Ni^II^Cl

DFT calculations were performed to shed light on the origin of the difference in the reaction behavior of **L1**Ni^II^Cl and **L2**Ni^II^Cl with acetonitrile. We assume that the reaction is initiated by the addition of MeCN to **L**Ni^II^Cl, generating the intermediate **L** ^MeCN^NiCl, which is then protonated to form the final product [**L** ^MeCNH^NiCl]­[Cl]. Figure 3 presents the calculated free energy profile for the addition reaction step. The activation energy of **L1**Ni^II^Cl was calculated to be 24.8 kcal/mol, much higher than 19.3 kcal/mol of **L2**Ni^II^Cl. This result is consistent with experimental observations that the latter can react with acetonitrile, while the former cannot. The distortion-interaction analysis reveals that, in fact, the binding energy between MeCN and Ni^II^ complex in **TS**_**L1**_ (−18.3 kcal/mol) is more negative than that in **TS**_**L2**_ (−12.1 kcal/mol). However, the strain energy of **TS**_**L1**_ (16.3 + 18.8 kcal/mol) is considerably higher than that of **TS**_**L2**_ (14.0 + 10.3 kcal/mol), resulting in **TS**_**L1**_ possessing a higher energy than **TS**_**L2**_. In other words, the longer arm length of **L2**Ni^II^Cl provides a larger geometric flexibility, which in turn leads to a lower activation energy. Furthermore, the reaction of **L1**Ni^II^Cl involves a forbidden singlet–triplet transition, which may be another reason why this complex cannot react with acetonitrile.

**Figure 3:**
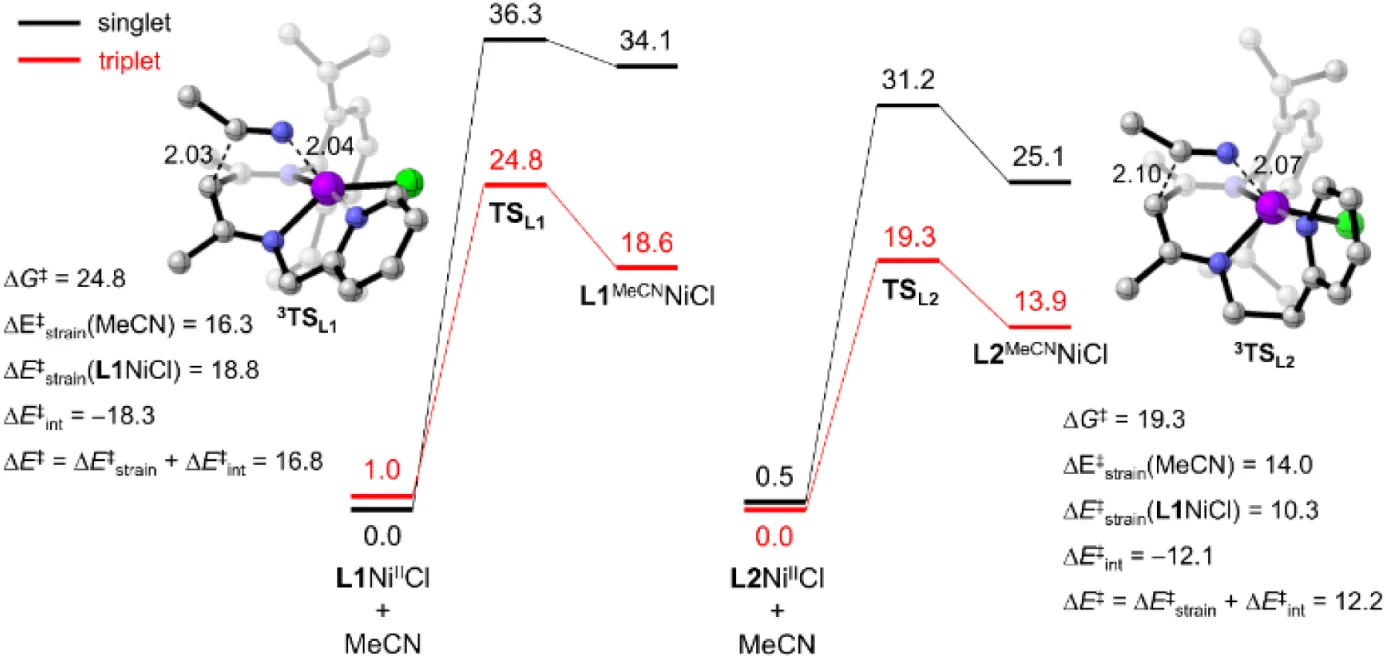
Free energy profiles of the addition reactions of acetonitrile with **L1**Ni^II^Cl and **L2**Ni^II^Cl. The optimized geometries of the transition states are given along with the results of the distortion-interaction analysis. The energy unit is kcal/mol.

### Preparation and Characterization of Nickel­(II)-Hydroxyl Complexes

To probe the influence of ligand architecture on hydroxide coordination and nuclearity, the reactivity of **L1**Ni^II^Cl and **L2**Ni^II^Cl toward KOH was examined in THF (Scheme 1). Treatment of **L1**Ni^II^Cl with KOH afforded the green mononuclear complex **L1**NiOH in good yield.^41^ The diamagnetic nature of **L1**NiOH was evident from its well-resolved ^1^H NMR spectrum in C_6_D_6_, which displays characteristic signals of the β-diketiminate ligand and a diagnostic upfield-shifted resonance at −4.73 ppm assigned to the Ni–OH proton, a hallmark of terminal metal-hydroxyl species (Figure S6).^53–55^ FT-IR spectroscopy further confirmed the presence of the hydroxide ligand, showing a sharp O–H stretch at 3637 cm^–1^, that shifts to 2680 cm^–1^ upon deuterium exchange, consistent with an O–D vibration (Figure S26). X-ray diffraction revealed a mononuclear square-planar nickel­(II) center coordinated by three nitrogen atoms from the β-diketiminate ligand and one terminal hydroxide (Figure 4a). Although the crystal structure of **L1**NiOH has been reported previously,^41^ it is discussed here to provide a direct structural comparison with **L2**Ni­(μ–OH)_2_Ni**L2** and to highlight the influence of pyridyl arm length on hydroxide coordination mode and nuclearity. Selected bond lengths and angles for **L1**NiOH and **L2**Ni­(μ–OH)_2_Ni**L2** are summarized in Table 2 for comparison.

**Figure 4:**
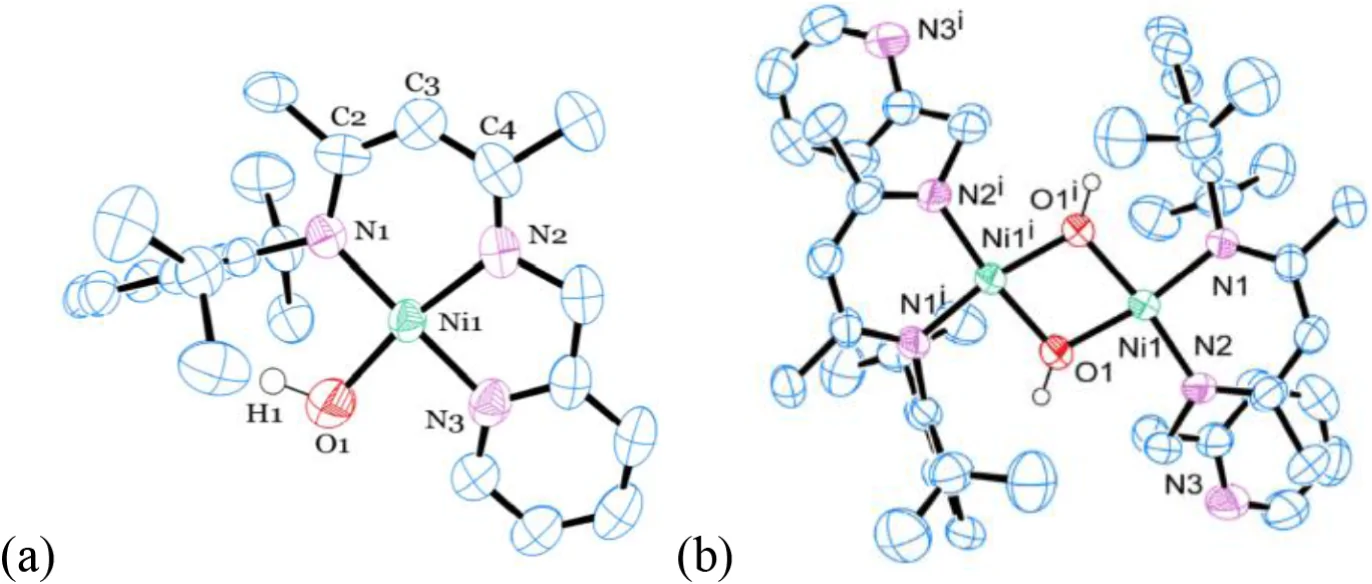
ORTEP drawings of (a) **L1**NiOH^41^ and (b) **L2**Ni­(μ–OH)_2_Ni**L2**. Thermal ellipsoids are drawn at the 50% probability level; hydrogen atoms are omitted for clarity. The structure of **L1**NiOH was reported previously.^41^

In marked contrast, the reaction of the longer-arm complex **L2**Ni^II^Cl with KOH under identical conditions yielded the green dimeric species **L2**Ni­(μ–OH)_2_Ni**L2** (Scheme 1). Crystallographic data revealed that each nickel center adopts square planar geometry, ligated by two nitrogen atoms from the β-diketiminate framework and two bridging hydroxide ligands. The pendant pyridine arms of the two ligands are oriented in opposite directions across the dimer, highlighting the conformational flexibility enabled by the extended ethyl linker in the six-membered chelate ring (Figure 4b). The complex is diamagnetic, consistent with low-spin d^8^ Ni­(II) in square-planar environments. These results demonstrate that extension of the pyridyl chelating arm profoundly alters the coordination behavior toward hydroxide. The shorter-arm ligand **L1** supports a mononuclear terminal hydroxide complex, whereas the longer-arm ligand **L2** favors dimerization with bridging hydroxides. This ligand-dependent switch in nuclearity illustrates how subtle changes in chelate ring size and steric profile can dictate the aggregation state and structural motifs of nickel-hydroxide species.

**Table 2:** Selected Bond Lengths (Å) and Angles (Degrees) for **L1**NiOH and **L2**Ni­(μ–OH)_2_Ni**L2**

**L1**NiOH		**L2**Ni(μ–OH)_2_Ni**L2**	
N(1)–Ni(1)	1.883(2)	N(1)–Ni(1)	1.8696(16)
N(2)–Ni(1)	1.882(2)	N(2)–Ni(1)	1.8808(17)
N(3)–Ni(1)	1.909(2)	O(1)–Ni(1)	1.8696(16)
O(1)–Ni(1)	1.854(2)	O(2)–Ni(1)	1.8716(17)
N(1)–Ni(1)–N(3)	175.61(9)	N(1)–Ni(1)–O(1)	169.11(7)
N(2)–Ni(1)–O(1)	169.63(9)	N(2)–Ni(1)–O(1)	172.07(7)

## Conclusions

This work demonstrates that the subtle extension of the pyridyl chelating arm in unsymmetrical *N*-aryl-*N′*-alkylpyridyl β-diketiminate ligands exerts profound control over the geometry, spin state, and reactivity of nickel­(II) and nickel­(I) complexes. The shorter-arm ligand H**L1** consistently enforces square-planar coordination in Ni­(II), yielding diamagnetic, low-spin complexes (e.g., **L1**Ni^II^Cl and **L1**NiOH). In contrast, the longer-arm ligand H**L2** induces significant distortion, producing the rare paramagnetic seesaw geometry in **L2**Ni^II^Cl and enabling dimerization to **L2**Ni­(μ–OH)_2_Ni**L2** upon hydroxide coordination. Reduction to Ni­(I) further amplifies this ligand dependence: **L1**Ni^I^ adopts a T-shaped geometry, while **L2**Ni^I^ favors Y-shaped coordination, with EPR spectra and crystallographic out-of-plane displacements (0.018 Å vs 0.099 Å) revealing ligand-modulated spin density and electronic structure.

Most strikingly, the extended arm in **L2**Ni^II^Cl confers exceptional reactivity toward alkyl nitriles, triggering slow but clean activation and rearrangement of the β-diketiminate backbone. This process (absent in the shorter-arm analogue) affords the five-coordinate high-spin adduct [**L2** ^MeCNH^NiCl]­[Cl] and provides compelling evidence for nitrile-induced noninnocent behavior of the β-diketiminate framework, representing an unprecedented transformation in nickel β-diketiminate chemistry. Collectively, the results establish that pyridyl chelating ring size (five- vs six-membered) and ligand flexibility serve as powerful handles for tuning nickel coordination geometry, spin multiplicity, nuclearity, and ligand-centered reactivity. This level of control offers a rational blueprint for designing nickel complexes with tailored electronic properties and enhanced reactivity profiles, with potential implications for bioinspired catalysis, small-molecule activation, and metalloenzyme model systems.

## Experimental Section

All experiments were under a purified dinitrogen atmosphere inside a glovebox or using the standard Schlenk line techniques. Chemical reagents were purchased from Sigma-Aldrich Co. Ltd., Lancaster Chemicals Ltd., or Fluka Ltd. Glassware was dried at 150 °C overnight before use. H**L1**,^26^ H**L2**,^26^ KC_8_ (potassium graphite),^56^ and NiCl_2_(2,4-lutidine)_2_,^57,58^ **L1**Ni^II^Cl,^41^ **L1**Ni^I^,^41^ **L1**NiOH^41^ were prepared according to the literature procedures. All solvents were dried over sodium (hexane, pentane, THF, and toluene) or CaH_2_ (d-benzene, CH_2_Cl_2_, and CH_3_CN) and then thoroughly degassed before use. UV–vis spectra were recorded on an Agilent 8453 spectrometer adapted with a Unisoku USP-203 Cryostat. ^1^H NMR and ^13^C NMR spectra were recorded using a Varian Gemini-200 NMR or a JEOL-400 NMR instrument. ESI mass spectra were collected on a Waters ZQ 4000 mass spectrometer. High-resolution mass spectra (HR-MS) were recorded on a Thermo Fisher Orbitrap Exploris 120 mass spectrometer equipped with an electrospray ionization (ESI) source. EPR spectrum were recorded on an EMXplus-10/12/P/L SYSTEM and EMXnano BENCH-TOP SYSTEM spectrometer.

### Preparation of Nickel­(II) Chloride Complex

**L2Ni** ^**II**^ **Cl:** NiCl_2_(2,4-lutidine)_2_ (2.6271 g, 7.59 mmol) was added to a solution of H**L2** (1.971 g, 5.42 mmol) in 20 mL THF, and the mixture was stirred for 30 min. A solid of NaOMe (0.293 g, 5.42 mmol) was added to the mixture, and the mixture was stirred for another 12 h. The solution was filtered and reduced to 10 mL under vacuum. The black residue was extracted with hexane (80 mL). After precipitation occurred, it was collected and dried under vacuum to yield a black solid with a hint of red, **L2**Ni^II^Cl (1.658 g, 3.632 mmol, 67%). A single crystal of **L2**Ni^II^Cl was obtained by cooling the saturated THF solution to −20 °C for 3 days. ^1^H NMR (C_6_D_6_, 400 MHz, 298 K, δ):85.97, 22.61, 21.65, 21.17, 12.91, 10.21, 4.25, 2.33, −2.09, −7.31, −23.35, −28.65, −61.98. μ_*eff*_ (C_6_D_6_, Evans method): 2.84 ± 0.2 μ_B_. μ_*eff*_ (SQUID): 2.571 μ_B_. UV–vis (toluene, 298 K, ε in M^–1^cm^–1^): 334 (10984), 392 (4844), 555 (420), 632 (168). (ESI)-HRMS: Calcd for C_24_H_32_N_3_NiCl [**L2**NiCl + H]^+^: 456.1717, found: 456.1711.

### Preparation of Nickel­(I) Complex

**L2Ni** ^**I**^: Potassium graphite (KC_8_) (0.106 g, 0.920 mmol) was added to a solution of **L2**Ni^II^Cl (0.300 g, 0.657 mmol) in 20 mL THF. The solution turned from reddish-black to wine red immediately, and the mixture was stirred for 2 h. The solvent was removed under vacuum, and the residue was extracted with 100 mL of pentane. The solution was filtered through a 0.22 μm syringe filter, and the filtrate was evaporated to dryness under vacuum to yield a deep red solid of **L2**Ni^I^ (0.193 g, 0.460 mmol, 70%). Single crystal **L2**Ni^I^ was obtained by cooling the saturated pentane solution at −20 °C for 1 day. UV–vis (toluene, 298 K, ε in M^–1^cm^–1^): 311 (11999), 389 (2830), 406 (2514), 448 (1295), 569 (1637). EPR (toluene, 100 K): g_1_ = 2.349, g_2_ = 2.141, g_3_ = 2.042.

### Preparation of Acetonitrile-Coordinated Nickel Complex

**[L2** ^**MeCNH**^ **NiCl]­[Cl]**: Dissolve **L2**Ni^II^Cl (0.3 g, 0.657 mmol) in 7 mL of acetonitrile. Leave the solution in a −20 °C refrigerator for 1 week to grow light blue crystals. Wash with 10 mL of benzene to yield the light blue solid of [**L2** ^MeCNH^NiCl]­[Cl] (0.11 g, 0.206 mmol, 33%). ^1^H NMR (CD_3_CN, 400 MHz, 298 K, δ): 56.81, 50.40, 21.52, 16.99, 16.25, 4.71, 3.57, 2.12, 1.92, 1.27, −3.18, −7.42, −14.95, −19.20, −25.11. μ_*eff*_ (CD_3_CN, Evan’s method): 2.86 ± 0.2 μ_B_. UV–vis (MeCN, 298 K, ε in M^–1^cm^–1^): 256 (8137), 335 (681), 626 (25). (ESI)-HRMS: Calcd for C_26_H_35_N_4_NiCl **[L2** ^MeCNH^NiCl]^**+**^: 497.1982, found: 497.1977.

### Preparation of Nickel-Hydroxyl Complex

**L2Ni­(μ–OH)**_**2**_ **NiL2**: Potassium hydroxide (0.025 g, 0.438 mmol) was added to a solution of **L2**Ni^II^Cl (0.200 g, 0.438 mmol) in 30 mL THF. The solution gradually turned from dark red to green, and the mixture was stirred for 10 min. The solvent was removed under vacuum, and the residue was extracted with 80 mL of diethyl ether. The solution was filtered through a 0.22 μm syringe filter, and the filtrate was evaporated to dryness under vacuum to yield a green solid of **L2**Ni­(μ–OH)_2_Ni**L2** (0.14 g, 0.320 mmol, 72.8%). Single crystals of **L2**Ni­(μ–OH)_2_Ni**L2** were obtained by cooling the saturated diethyl ether solution to −35 °C for 3 days.^1^H NMR (C_6_D_6_, 400 MHz, 298 K, δ): 9.04 (d, 1H, Py_1_), 7.33 (s, 3H, Ar–H), 6.68 (t, 1H, Py_2_), 6.53 (t, 1H, Py_3_), 6.25 (d, 1H, Py_4_), 4.88 (s, 1H, backbone-CH), 4.07 (septet, 2H, ArCH­(CH_3_)_2_), 3.76 (t, 2H, CH_2_Py), 3.26 (t, 2H, CH_2_Py), 2.04 (d, 6H, ArCH­(CH_3_)_2_), 1.70 (s, 3H, backbone-CH_3_), 1.50 (s, 3H, backbone-CH_3_), 1.35 (d, 6H, ArCH­(CH_3_)_2_), −5.03 (s, 1H, OH). UV–vis (toluene, 298 K, ε in M^–1^ cm^–1^): 321 (6020), 382 (3990), 454 (sh, 475), 609 (47). FT-IR (KBr): ν­(O–H) = 3625, 3600 cm^–1^.

## Supplementary Material





## Notes

Spectroscopic; crystallographic; computational details; Cartesian (XYZ)
Coordinates of the computed species (PDF)
