# Supplementary material for: Ligand Arm Length Governs Geometry, Spin State, and Reactivity in Unsymmetrical β‑Diketiminato Nickel(II) and Nickel(I) Complexes
Source: Inorg Chem. 2026 Aug 28;65(36):21108–16. doi: 10.1021/acs.inorgchem.6c03078 (PMC13580181; doi:10.1021/acs.inorgchem.6c03078)
Supplement: Supplementary file 1 [file ic6c03078_si_002.pdf]

Supporting Information

For

Ligand Arm Length Governs Geometry, Spin State, and Reactivity in Unsymmetrical β-Diketiminato Nickel(II) and Nickel(I) Complexes

Tzu-Hsien Yang[†,#] Si-Hong Chen[†,#] Yu-Ting Chu[‡] Ming-Li Tsai[§] Hsing-Yin Chen*[,†]
Sodio C. N. Hsu*[,†,‖]

[†]Department of Medicinal and Applied Chemistry, College of Life Sciences, Kaohsiung Medical University, Kaohsiung 80708, Taiwan

[‡]International PhD Program for Science, National Sun Yat-Sen University, Kaohsiung 80424, Taiwan

[§]Department of Chemistry, National Sun Yat-Sen University, Kaohsiung 80424, Taiwan

[‖]Department of Medical Research, Kaohsiung Medical University Hospital, Kaohsiung 80708, Taiwan

Corresponding author: E-mail: sodiohsu@kmu.edu.tw, hychen@kmu.edu.tw

Table of Content

SI. Experimental Section **4**
X-ray Crystal Structure Determinations. 4
EPR Measurements. 4
SI. Computational Methods **4**
SI. NMR Spectra **5**
Figure S1. ^{1}H NMR spectrum of **L1**Ni^{II}Cl in C_6D_6 (400 MHz, 298 K). Solvent residual peaks are marked with an asterisk (*). 5
Figure S2. $^{13}C\{^1H\}$ NMR spectrum of **L1**Ni^{II}Cl in C_6D_6 (100.58 MHz, 298 K). Solvent residual peaks are marked with an asterisk (*). 5
Figure S3. ^{1}H NMR spectrum of **L2**Ni^{II}Cl in C_6D_6 (400 MHz, 298 K). 6
Figure S4. Variable-temperature NMR spectrum of **L2**Ni^{II}Cl (d^8-toluene, 400 MHz). 6
Figure S5. ^{1}H NMR spectrum of [**L2**^{MeCNH}NiCl][Cl] (CD_3CN, 400 MHz, 298 K). 7
Figure S6. ^{1}H NMR spectrum of **L1**NiOH (C_6D_6, 400 MHz, 298 K). 7
SI. Evans Method **8**
Figure S7. Evans method of **L2**Ni^{II}Cl. 8
Figure S8. Evans method of [**L2**^{MeCNH}NiCl][Cl]. 9
SI. Superconducting Quantum Interference Device (SQUID) **10**
Figure S9. SQUID data of **L2**Ni^{II}Cl. 10
SI. EPR Spectra **11**
Figure S10. EPR spectrum of **L1**Ni^I (toluene, 1 x 10^{-3} M, 100K). 11
Figure S11. EPR spectrum of **L2**Ni^I (toluene, 1 x 10^{-3} M, 100K). 11
SI. UV-vis Absorption Spectroscopy **12**
Figure S12. UV-vis absorption spectrum of **L1**Ni^{II}Cl (toluene,10^{-4} M). 12
Figure S13. UV-vis absorption spectrum of **L2**Ni^{II}Cl (toluene, 10^{-4} M). 13
Figure S14. Variable-temperature UV-vis spectrum of **L2**Ni^{II}Cl (toluene, 2 x 10^{-3} M). 13
Figure S15. UV-vis absorption spectrum of **L1**Ni^I (toluene, 1 x 10^{-4} M). 14
Figure S16. UV-vis absorption spectrum of **L2**Ni^I (toluene, 1.1 x 10^{-4} M). 14
Figure S17. UV-vis absorption spectrum of [**L2**^{MeCNH}NiCl][Cl] (acetonitrile, 10^{-4} M). 15
Figure S18. UV-vis absorption spectrum of **L1**NiOH (toluene, 10^{-4} M). 16
Figure S19. UV-vis absorption spectrum of **L2**Ni(μ-OH)$_2$Ni**L2** (toluene, 10^{-4} M). 17
SI. Mass Spectrometry **18**
Figure S20. ESI-MS spectrum of **L1**Ni^{II}Cl (in acetonitrile). 18
Figure S21. HR-MS spectrum of **L2**Ni^{II}Cl (in THF). 18
Figure S22. HR-MS spectrum of [**L2**^{MeCNH}NiCl]$^+$ (in acetonitrile). 19
Figure S23. ESI-MS spectrum of [**L2**^{MeCNH}NiCl]$^+$, [**L2**^{EtCNH}NiCl]$^+$ and [**L2**$^{\textit{ipr}CNH}$NiCl]$^+$ (in acetonitrile). 19
Figure S24. ESI-MS spectrum of **L1**Ni^{II}Cl in acetonitrile in the presence of HCl/Et_2O (in acetonitrile). 20

Figure S25. ESI-MS spectrum of independently synthesized [**L1**Ni(MeCN)]$^+$[CF_3SO_3]$^-$ in acetonitrile. 20
SI. FT-IR Spectra 21
Figure S26. FT-IR spectrum (KBr) of **L1**NiOH (top) and its deuteration product **L1**NiOD (bottom). 21
Figure S27. FT-IR spectrum (KBr) of **L2**Ni(μ-OH)$_2$Ni**L2**. 21
SI. Crystal Data 22
Table S1. Selected bond lengths (Å) and angles (degrees) for **L2**Ni^{II}Cl and **L2**Ni^{I}. 22
Table S2. Crystallographic data for complexes **L2**Ni^{II}Cl and **L2**Ni^{I}. 23
Table S3. Selected bond lengths (Å) and angles (degrees) for [**L1**Ni(MeCN)][CF_3SO_3] and [**L2**^{MeCNH}NiCl][Cl]. 24
Table S4. Crystallographic data for complexes [**L1**Ni(MeCN)][CF_3SO_3] and [**L2**^{MeCNH}NiCl][Cl]. 25
Table S5. Selected bond lengths (Å) and angles (degrees) for [H**L2**H][Cl] and **L2**Ni(μ-OH)$_2$Ni**L2**. 26
Table S6. Crystallographic data for complexes [H**L2**H][Cl] and **L2**Ni(μ-OH)$_2$Ni**L2**. 27
SI. References 28

SI. Experimental Section

X-ray Crystal Structure Determinations.

All single X-ray diffraction data were accumulated using Rigaku Oxford Diffraction single crystal X-ray diffractometers with Mo Kα radiation (l = 0.71073 Å). The data collection was executed using the CrysAlisPro 1.171.41.56a program. Cell refinement and data reduction were made with the CrysAlisPro 1.171.41.56a program. The structure was determined using the Olex2/ ShelXL program, refined using full-matrix least squares. All non-hydrogen atoms were refined anisotropically, whereas hydrogen atoms were placed at calculated positions and included in the final stage of refinement with fixed parameters.

EPR Measurements.

The low-temperature EPR spectra of the Ni complexes **L2**Ni^{I} dissolved in toluene solution were measured at 100 K via a Bruker EMX plus/EMX-nano spectrometer. In a typical experiment, 0.4 mL of a 2.2 mM solution of the **L2**Ni^{I} in toluene was introduced into a gas-tight EPR tube (Willmad 707-LPV-200M) under anaerobic conditions. The EPR tube was placed in a liquid nitrogen bath for 5 min to allow thermal equilibration prior to measurements. The experimental parameters: microwave frequency = 9.628 GHz, microwave power = 0.32 mW, modulation amplitude = 0.5 mT, modulation frequency = 100 kHz, received gain = 40, time constant = 1.28 ms for **L2**Ni^{I}; Spin Hamiltonian parameters of **L2**Ni^{I} were determined by fitting the spectrum using Aniso-SpinFit program installed in the instrument.

SI. Computational Methods

Geometry optimization and vibrational frequency calculations were carried out at the TPSSh/6-31+G(d) level of theory. Single point energy calculations at the TPSSh/Def2-TZVP level were performed to provide more accurate energies. The SMD solvation model[1] was used to simulate the solvent effect (acetonitrile). Since the parameters of the SMD solvation model were developed using the M05-2X/6-31G(d) method, this method was used to evaluate the solvation energy in this study. The Wertz correction was adopted to remedy the error in solution entropy caused by using gas-phase statistical thermodynamics formulas in standard quantum chemical calculations.[2-6] According to this approach, the absolute entropies of 1 M solutes and 19.15 M acetonitrile in acetonitrile solution were calculated by equations 1 and 2, respectively. All calculations were done using the Gaussian 16 program.[7]

$$S_s = 0.77S^{\circ}_{gas} - \left(3.543\ \text{cal}\cdot\text{mol}^{-1}\cdot\text{K}^{-1}\right) \quad (1)$$

$$S_s = 0.77S^{\circ}_{gas} - \left(9.409\ \text{cal}\cdot\text{mol}^{-1}\cdot\text{K}^{-1}\right) \quad (2)$$

SI. NMR Spectra

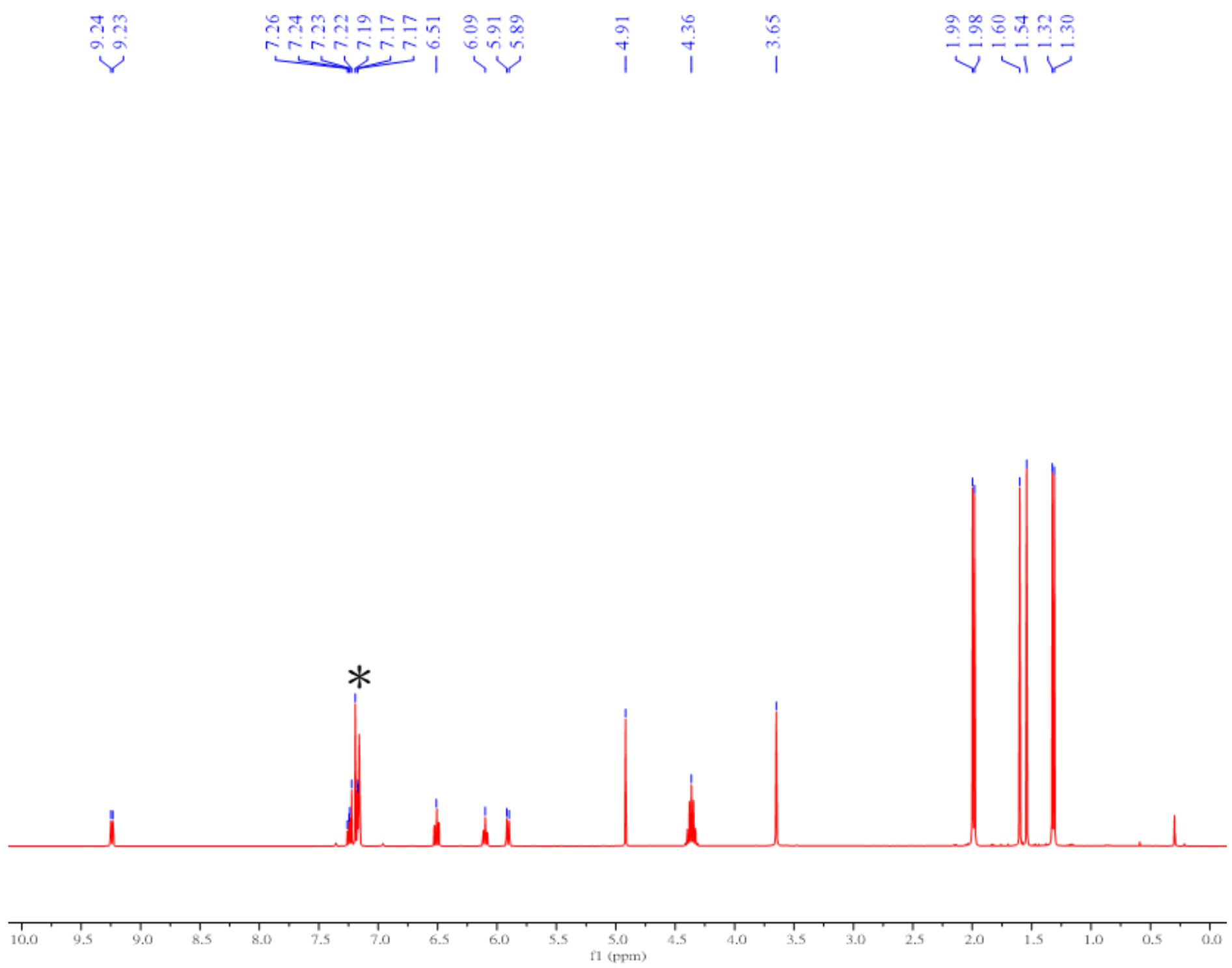

Figure S1. ^{1}H NMR spectrum of **L1**Ni^{II}Cl in C_6D_6 (400 MHz, 298 K). Solvent residual peaks are marked with an asterisk (*). The complex of **L1**Ni^{II}Cl was reported previously.[8]

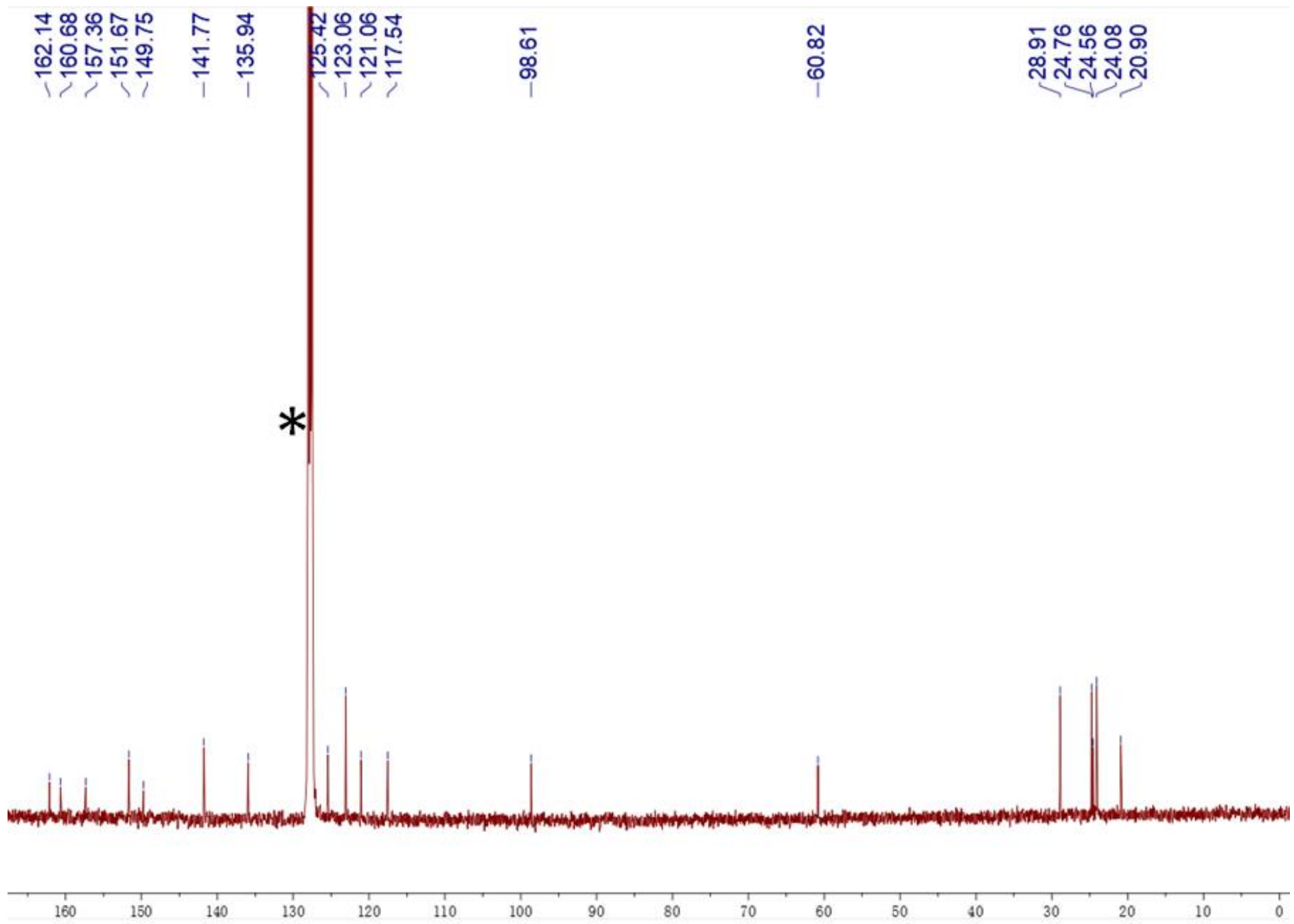

Figure S2. ^{13}C{^{1}H} NMR spectrum of **L1**Ni^{II}Cl in C_6D_6 (100.58 MHz, 298 K). Solvent residual peaks are marked with an asterisk (*). The complex of **L1**Ni^{II}Cl was reported previously.[8]

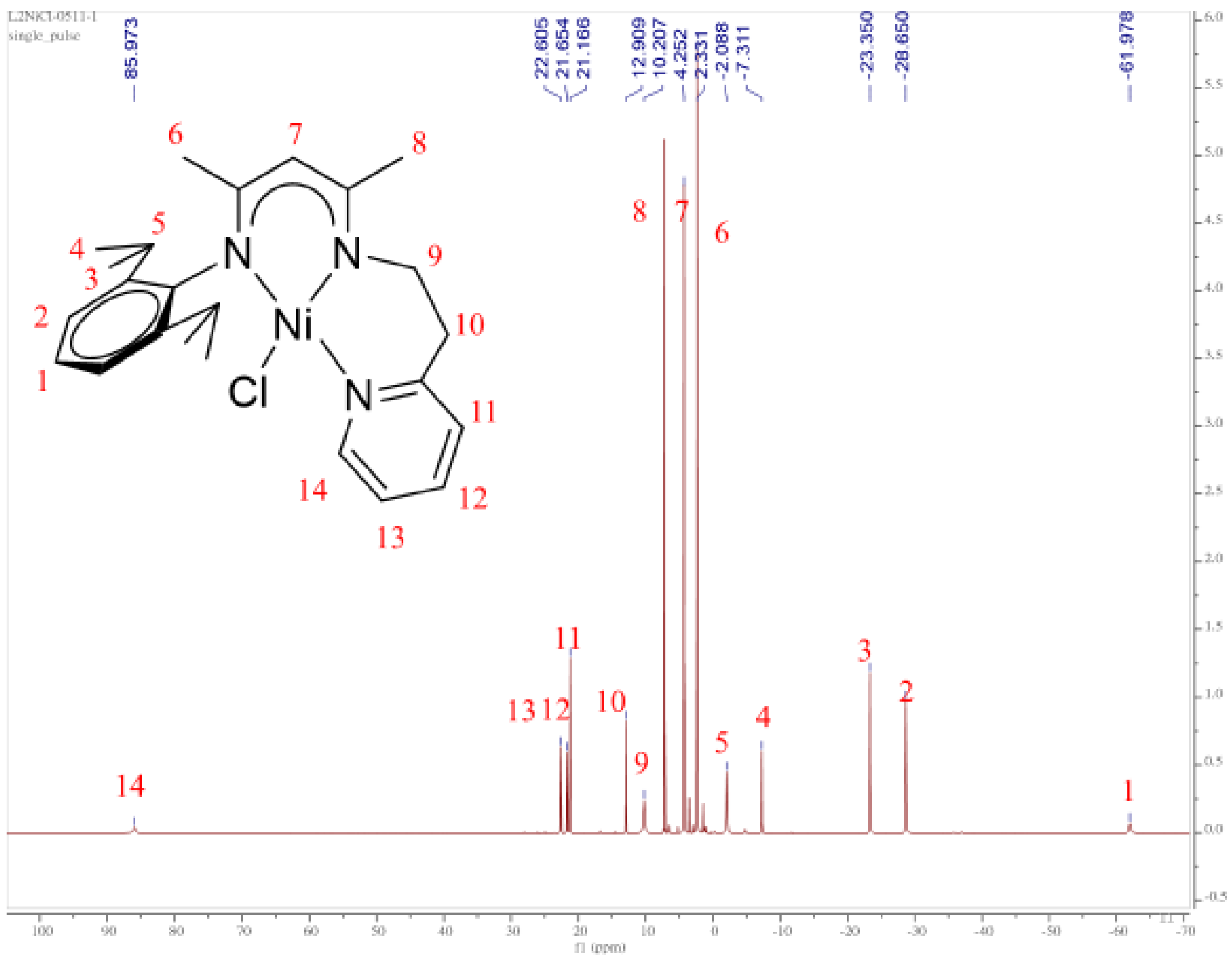

Figure S3. ^{1}H NMR spectrum of **L2**$Ni^{II}Cl$ in C_6D_6 (400 MHz, 298 K). The numbering is intended only to indicate the number of chemically distinct proton environments in the complex and does not imply definitive resonance assignments.

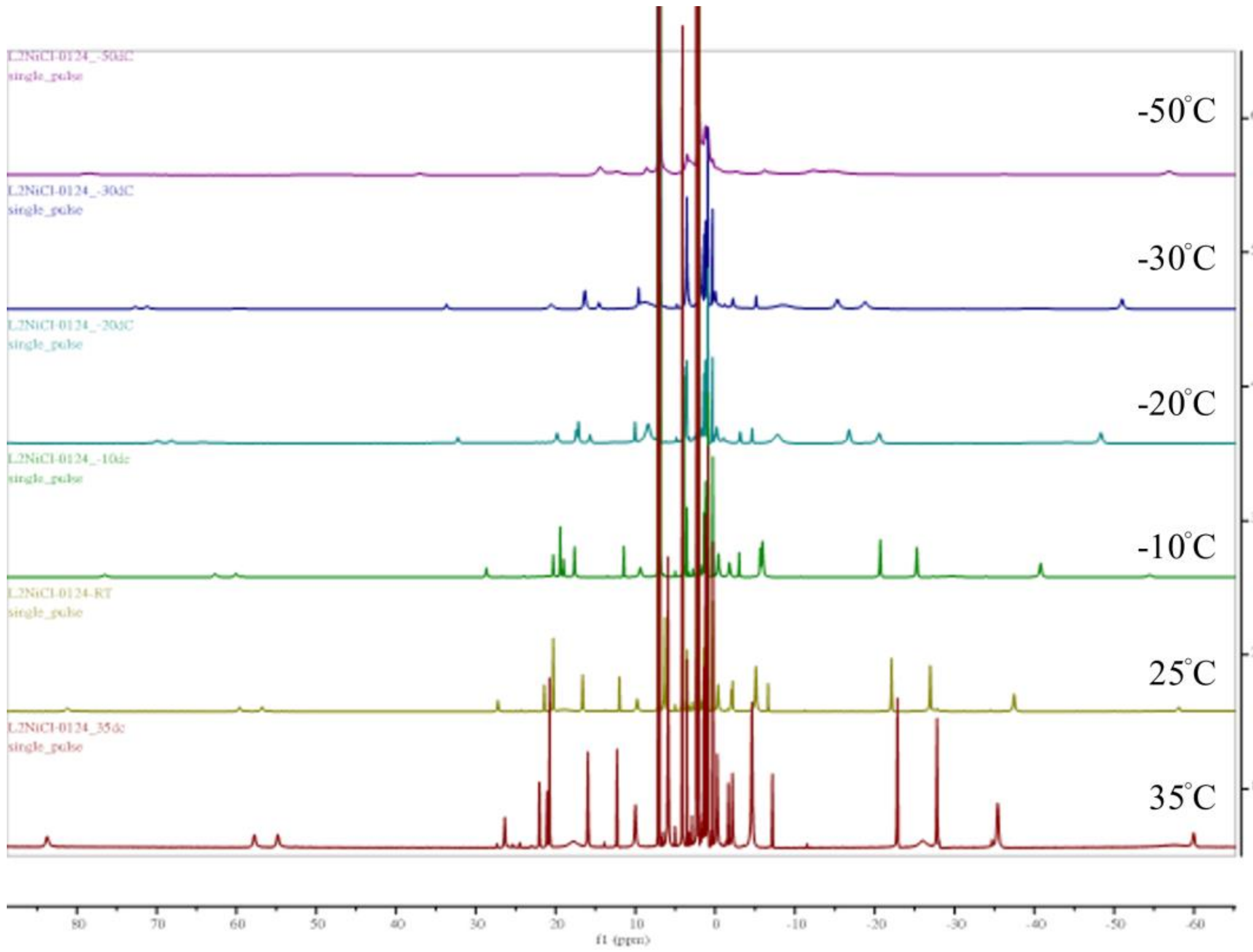

Figure S4. Variable-temperature NMR spectrum of **L2**$Ni^{II}Cl$ (d^8-toluene, 400 MHz).

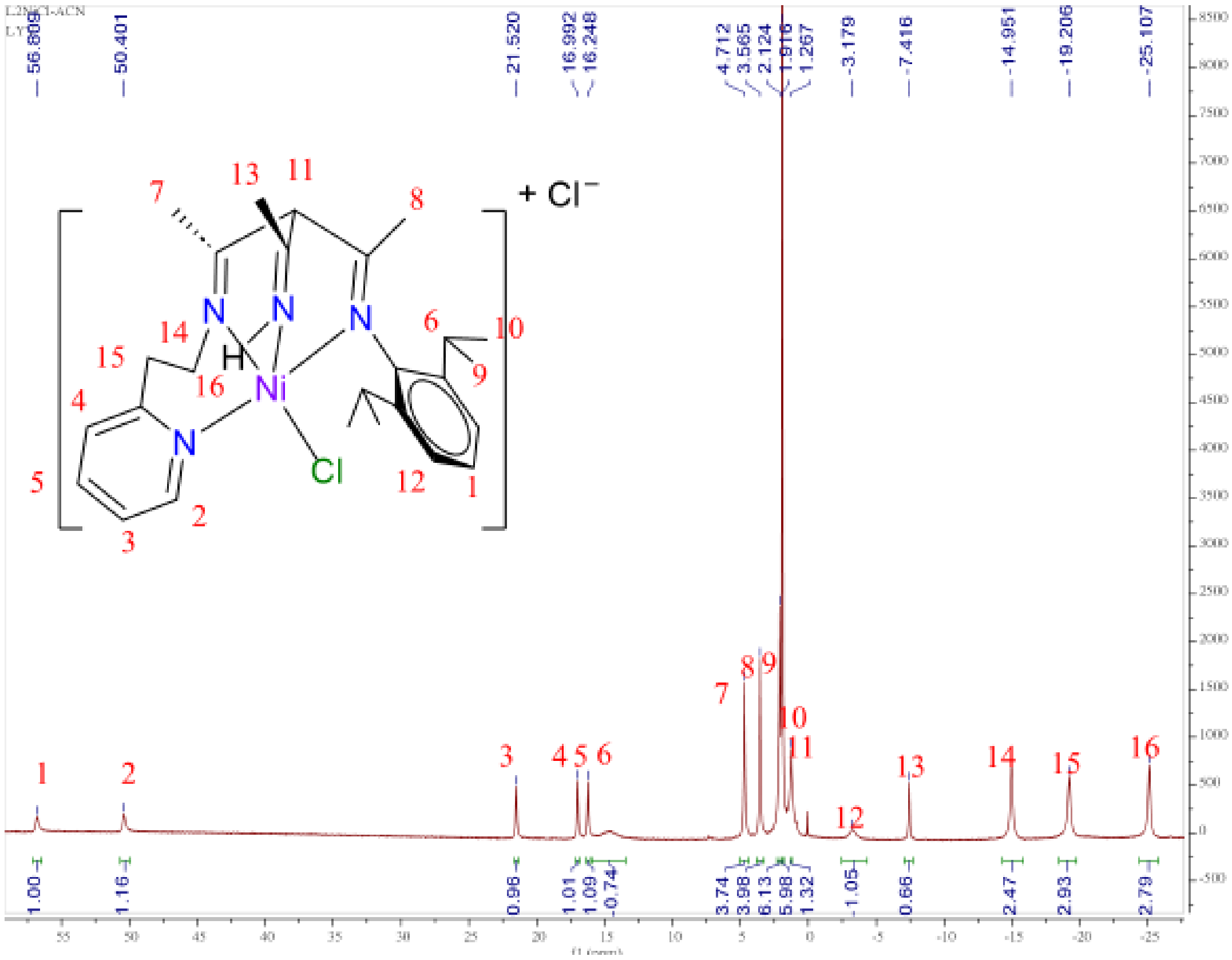

Figure S5. ^{1}H NMR spectrum of [**L2**^{MeCNH}NiCl][Cl] (CD_3CN, 400 MHz, 298 K).

The numbering is intended only to indicate the number of chemically distinct proton environments in the complex and does not imply definitive resonance assignments.

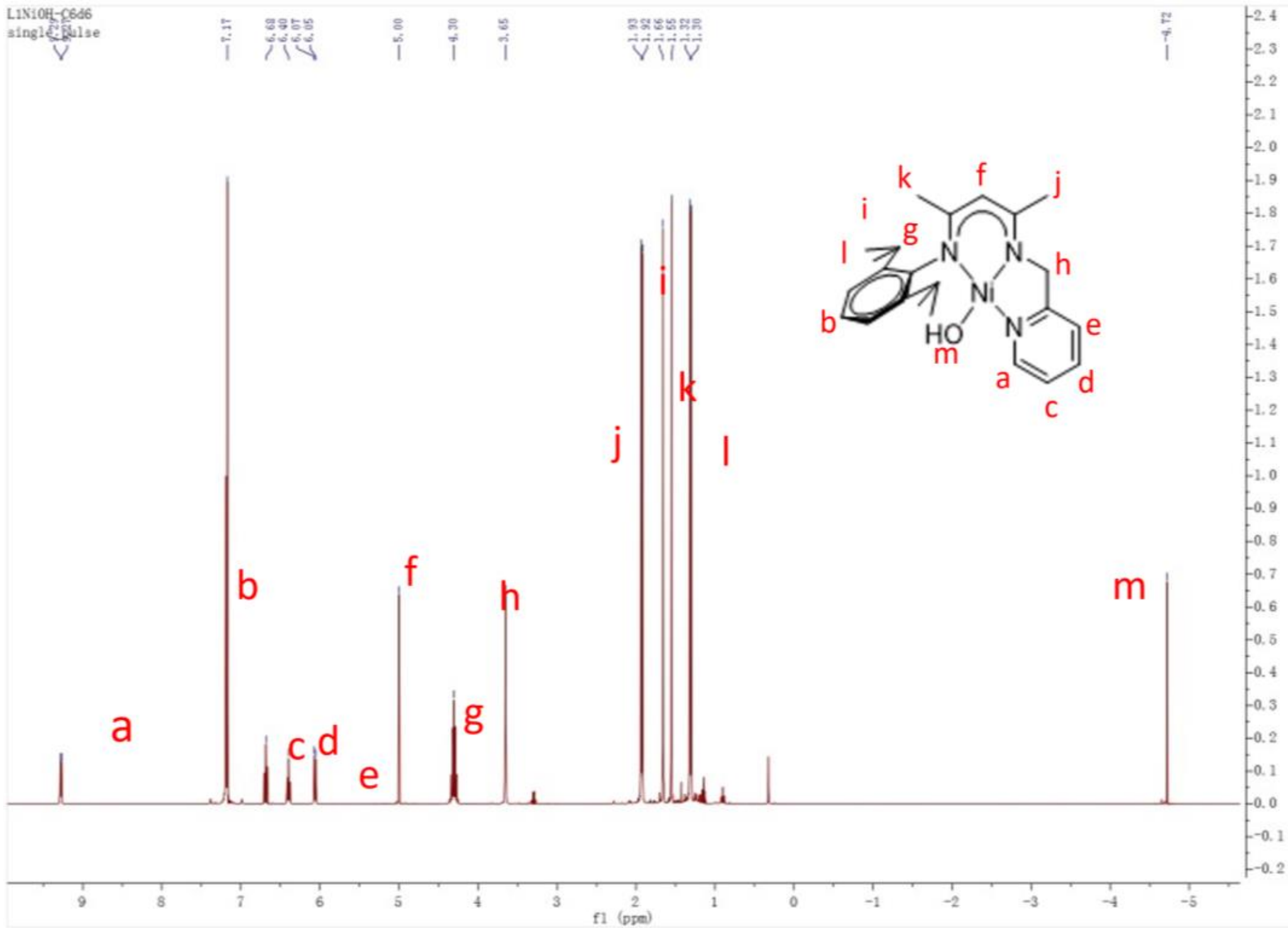

Figure S6. ^{1}H NMR spectrum of **L1**NiOH (C_6D_6, 400 MHz, 298 K). The complex of **L1**NiOH was reported previously.[8]

SI. Evans Method

Evans Method for L2Ni^{II}Cl

A capillary tube containing C_6D_6 with 2% THF as an internal standard was placed at the center of an NMR tube, while the outer tube contained **L2**Ni^{II}Cl dissolved in C_6D_6 with 0.5% THF. The ^{1}H NMR spectrum displayed two THF signals around 3.50 ppm, and the resulting chemical shifts were used to calculate the mass susceptibility of the sample (see **Figure S7**).

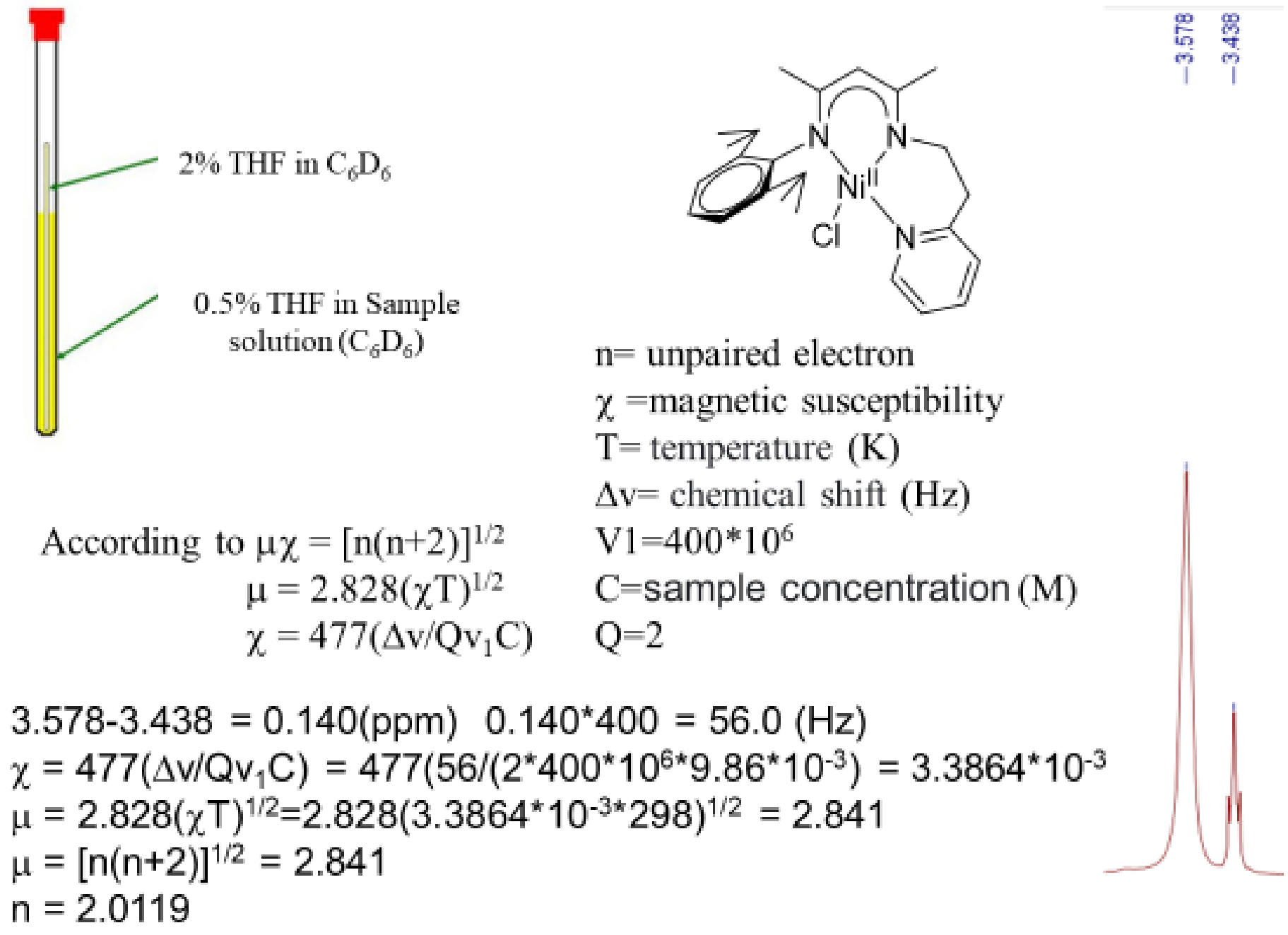

Figure S7. Evans method of **L2**Ni^{II}Cl.

Evans Method for [**L2**^{MeCNH}NiCl][Cl]

A capillary tube containing CD_3CN with 2% C_6H_6 as an internal standard was placed at the center of an NMR tube, while the outer tube contained [**L2**^{MeCNH}NiCl][Cl] dissolved in CD_3CN with 0.5% C_6H_6. The ^{1}H NMR spectrum displayed two benzene signals around 7.35 ppm, and the resulting chemical shifts were used to calculate the mass susceptibility of the sample (see **Figure S8**).

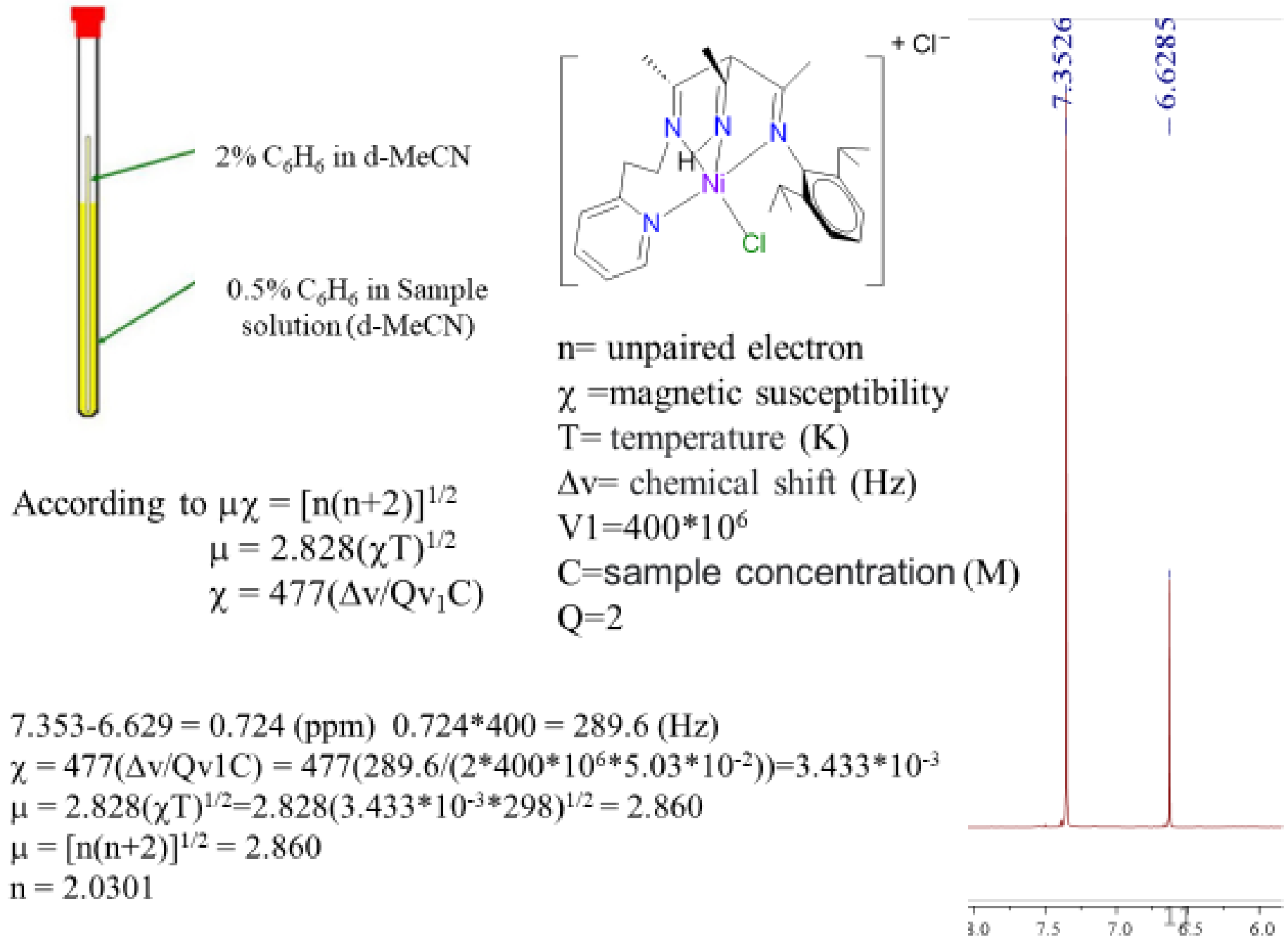

Figure S8. Evans method of [**L2**^{MeCNH}NiCl][Cl].

SI. Superconducting Quantum Interference Device (SQUID)

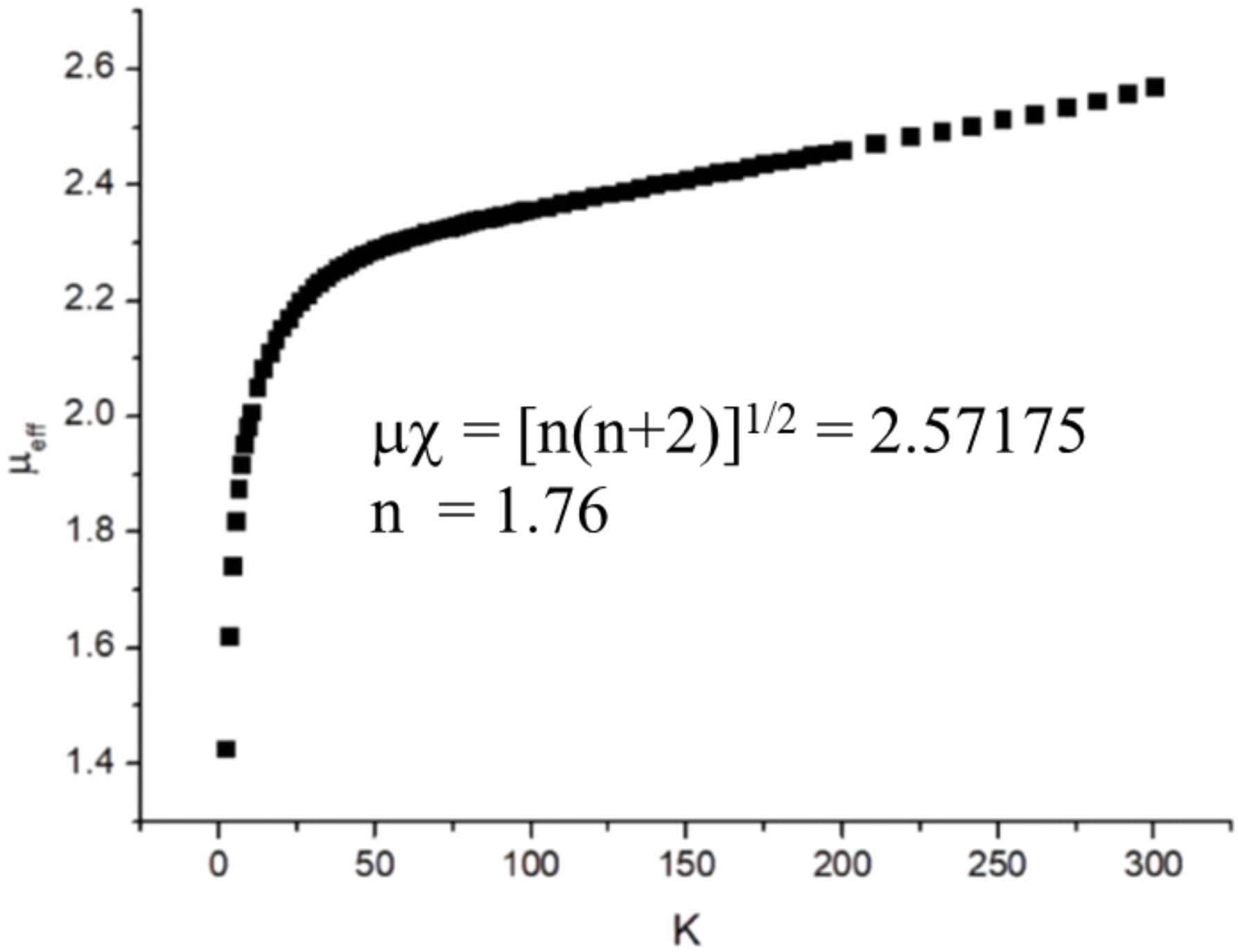

Figure S9. SQUID data of **L2**$Ni^{II}Cl$.

SI. EPR Spectra

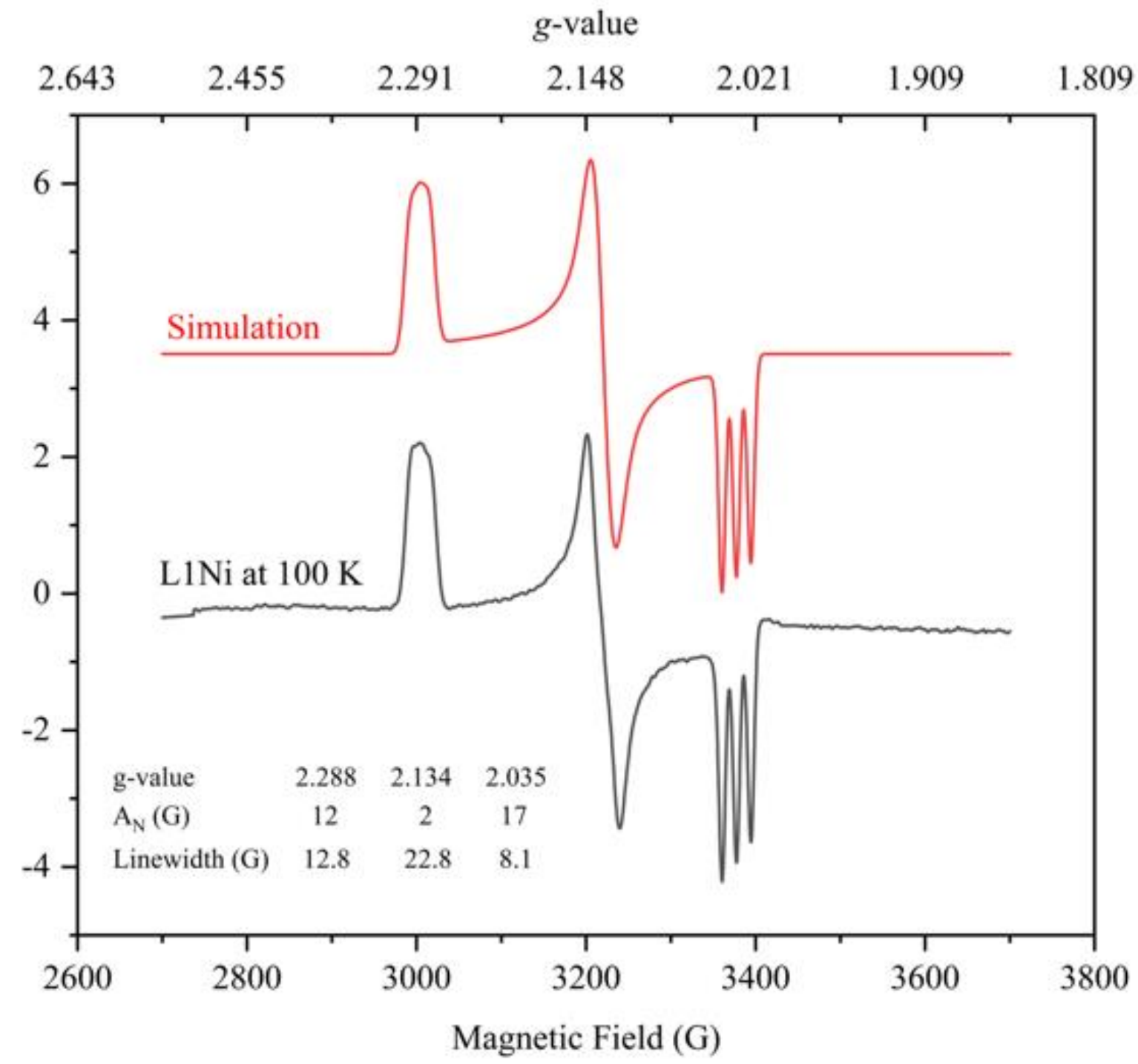

Figure S10. EPR spectrum of **L1**Ni^{I} (toluene, 1 x 10^{-3} M, 100 K). The complex of **L1**Ni^{I} was reported previously.[8]

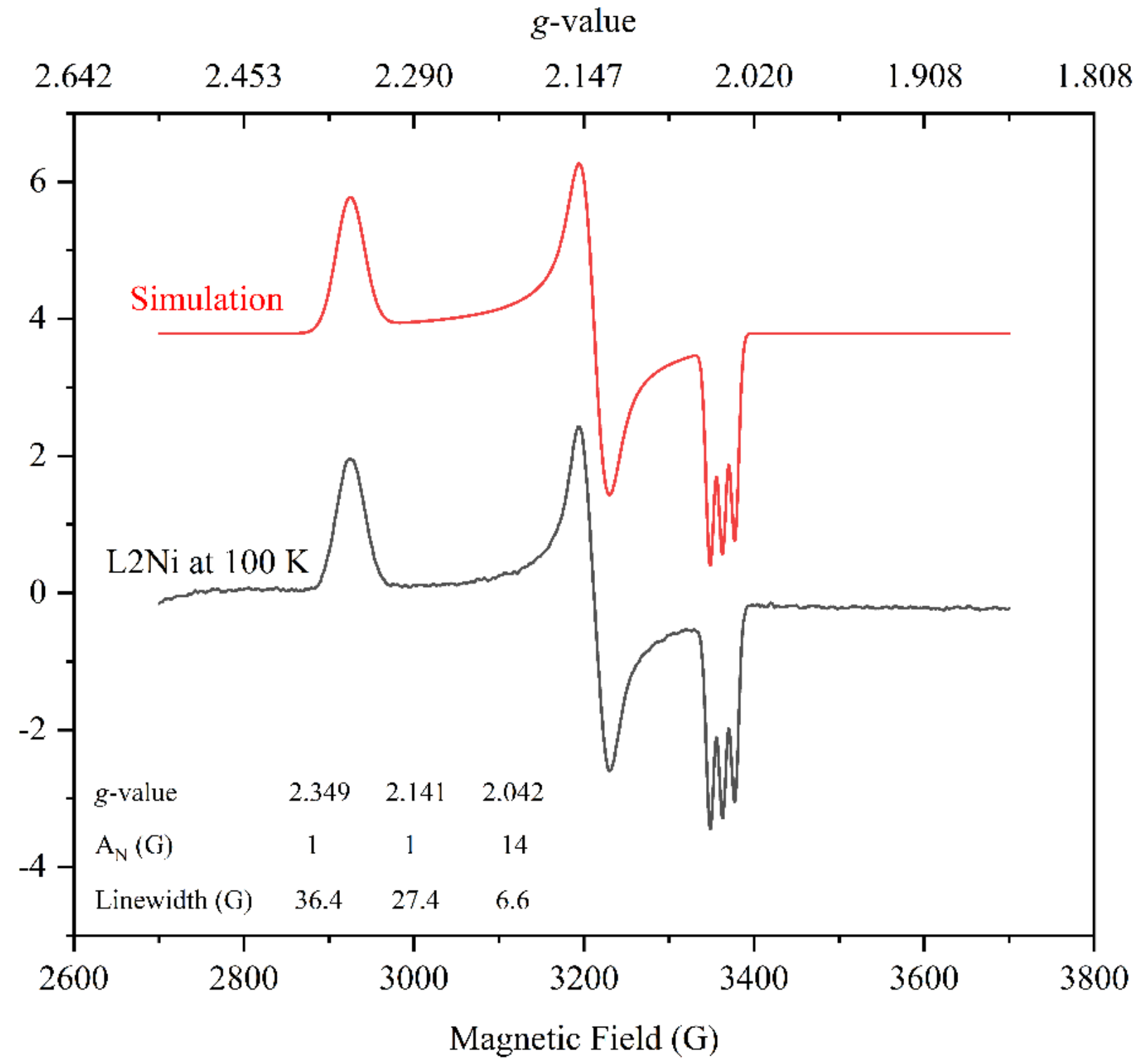

Figure S11. EPR spectrum of **L2**Ni^{I} (toluene, 1 x 10^{-3} M, 100 K).

SI. UV-vis Absorption Spectroscopy

Intense bands in the UV region (typically ε>1000-10000 $M^{-1}cm^{-1}$) are assigned as ligand-to-metal or metal-to-ligand charge transfer (CT) or ligand-centered π→π*/ n→π* transitions of the β-diketiminate framework. Weaker visible bands are d–d transitions. Square-planar (approximately centrosymmetric) low-spin Ni(II) complexes exhibit weak, Laporte-forbidden d-d bands (ε commonly < 100-200 $M^{-1}cm^{-1}$). Lower-symmetry geometries (seesaw, five-coordinate) relax these restrictions, producing higher ε values. High-spin configurations and three-coordinate Ni(I) species further modulate band intensities and energies. Based on these principles, the UV-vis spectra of the present nickel complexes are assigned and discussed below.

L1$Ni^{II}Cl$ (shorter-arm, square-planar, diamagnetic low-spin d^8)

UV-vis (toluene, 298 K, ε in $M^{-1}cm^{-1}$): 359 (6179; charge transfer), 386 (6625; charge transfer), 498 (128; d-d transition), 673 (45; d-d transition) nm.

The intense UV bands are characteristic LMCT transitions involving the β-diketiminate and pyridyl donors. The two weak visible bands are classic for square-planar Ni(II). Their low molar absorptivities are consistent with the near-centrosymmetric geometry ($\tau_4 \approx 0.10$) and diamagnetic ground state reported crystallographically. This spectrum matches the behavior expected for rigid five-membered chelate rings that enforce planarity.

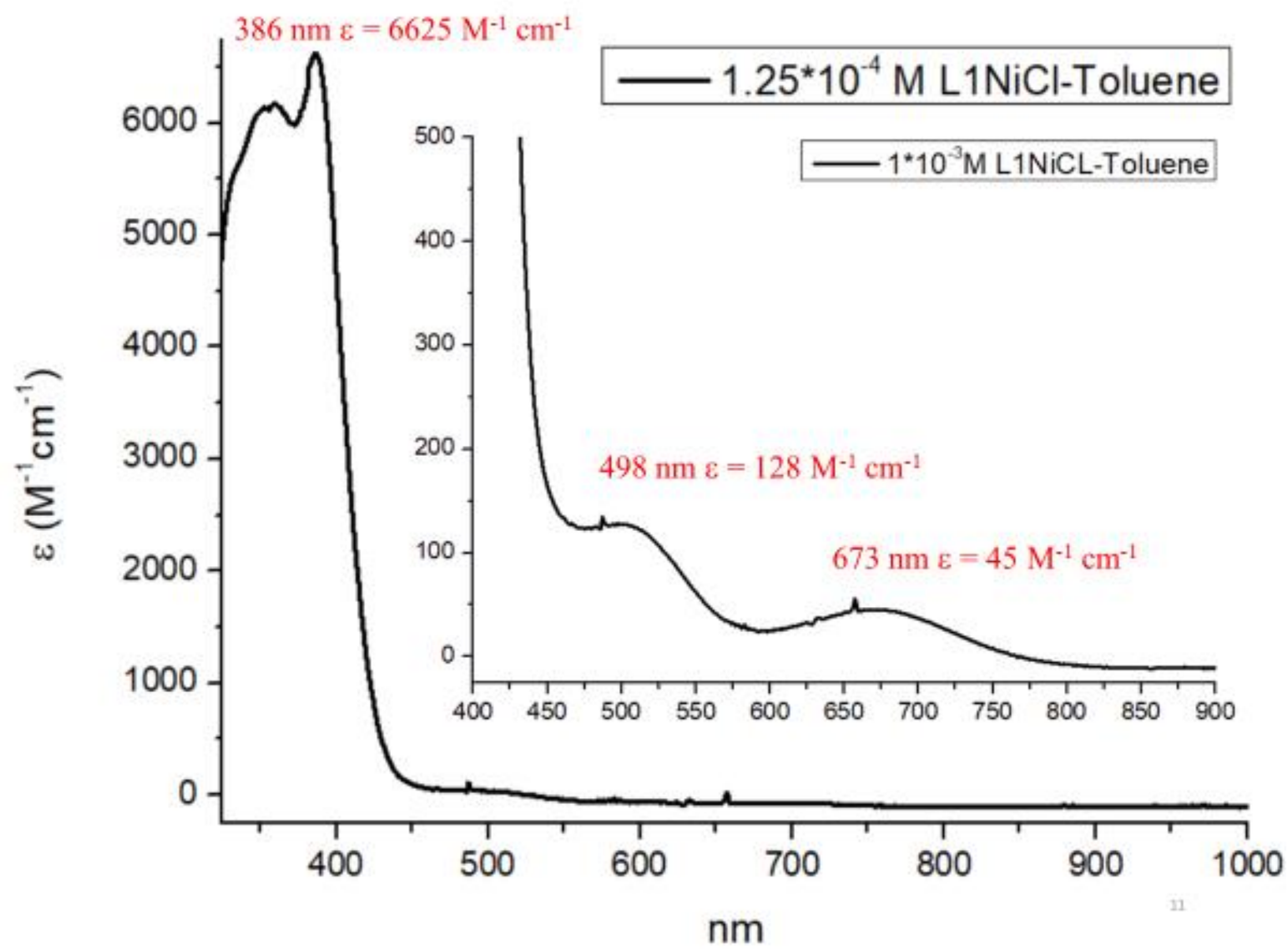

Figure S12. UV-vis absorption spectrum of **L1**$Ni^{II}Cl$ (toluene,10^{-4} M). The complex of **L1**$Ni^{II}Cl$ was reported previously.[8]

L2Ni^{II}Cl (longer-arm, distorted seesaw, paramagnetic high-spin)

UV-vis (toluene, 298 K, ε in $M^{-1}cm^{-1}$): 334 (10984; charge transfer), 392 (4844; charge transfer), 555 (420; d-d transition), 632 (168; d-d transition) nm.

The LMCT bands remain intense, but the d–d transitions are markedly stronger than in **L1**Ni^{II}Cl. This intensity increase is expected for the lower-symmetry seesaw geometry ($\tau_4 \approx 0.30$) imposed by the six-membered chelate ring; the absence of a strict center of inversion relaxes the Laporte selection rule. The magnetic data confirm the high-spin d^8 configuration that further contributes to the observed intensities. These spectral differences provide clear electronic evidence for the arm-length-dependent geometric switch.

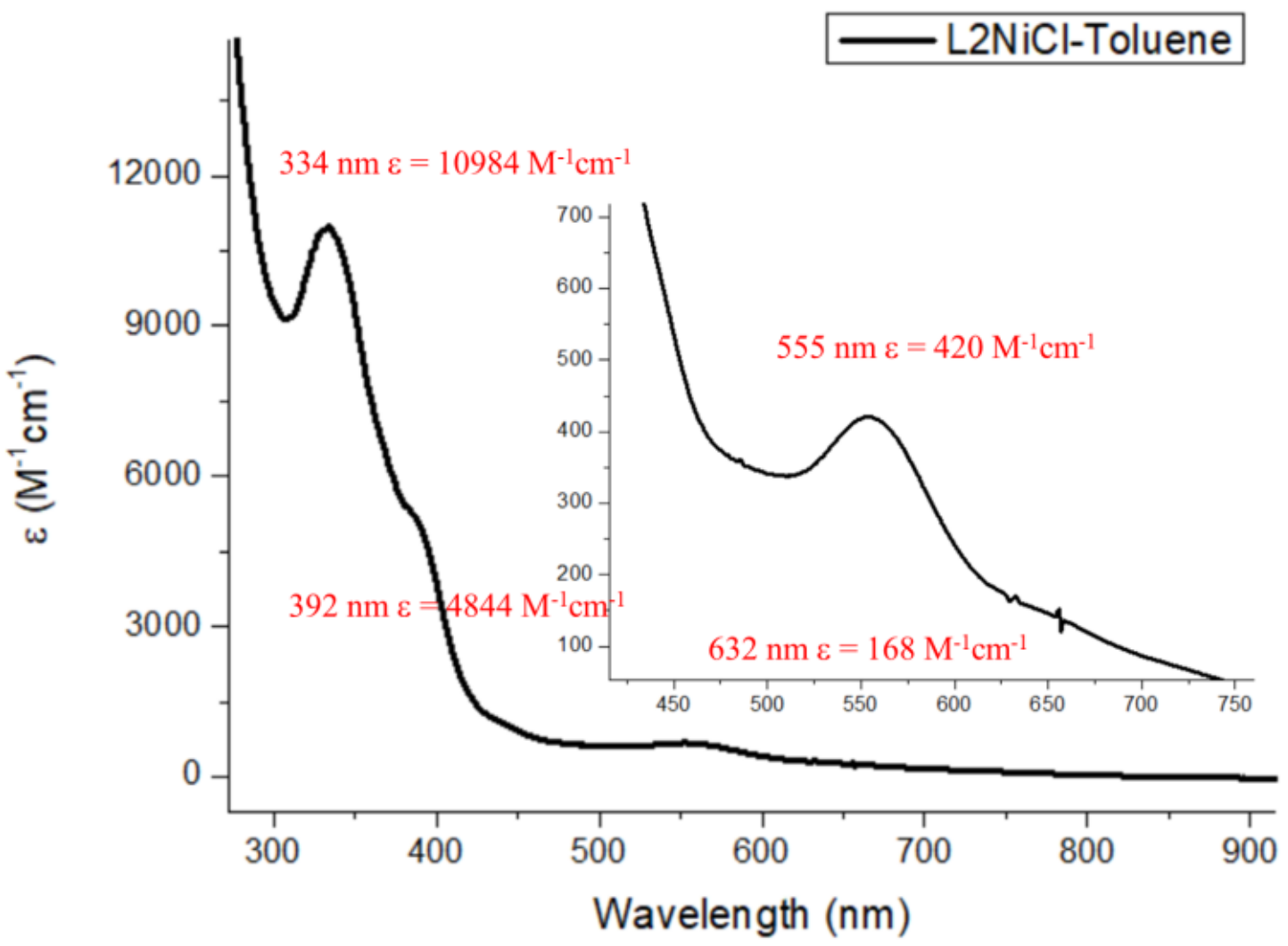

Figure S13. UV-vis absorption spectrum of **L2**Ni^{II}Cl (toluene, 10^{-4} M).

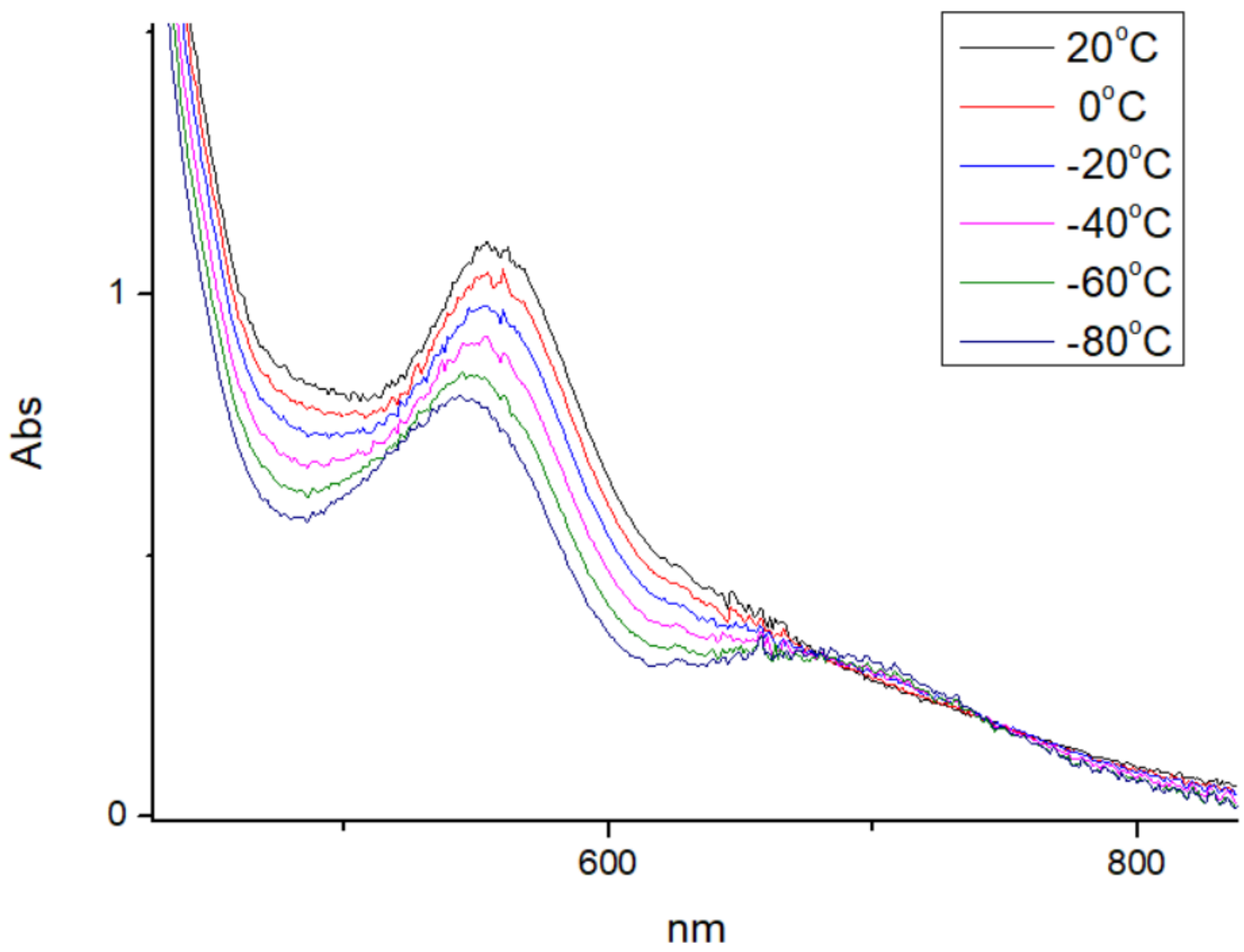

Figure S14. Variable-temperature UV-vis spectrum of **L2**Ni^{II}Cl (toluene, 2 x 10^{-3} M).

L1Ni^{I} **and L2**Ni^{I} (three-coordinate Ni(I), d^9)

Both species display relatively intense visible bands (especially the ~555-569 nm features with ε>1000). Three-coordinate Ni(I) complexes commonly show stronger absorptions than four-coordinate Ni(II) because the lower coordination number and d^9 configuration produce less strictly forbidden transitions and often involve significant metal-to-ligand or mixed charge-transfer character. The modest differences between the two spectra parallel the crystallographic distinction (T-shaped vs Y-shaped) and the small variation in EPR g_1 values, reflecting ligand-modulated spin density.

L1Ni^{I} (T-shaped) UV-vis (toluene, 298 K, ε in $M^{-1}cm^{-1}$): 305 (sh, 6471; charge transfer), 392 (3656; charge transfer), 411 (3293; d-d transition), 555 (1037; d-d transition) nm.

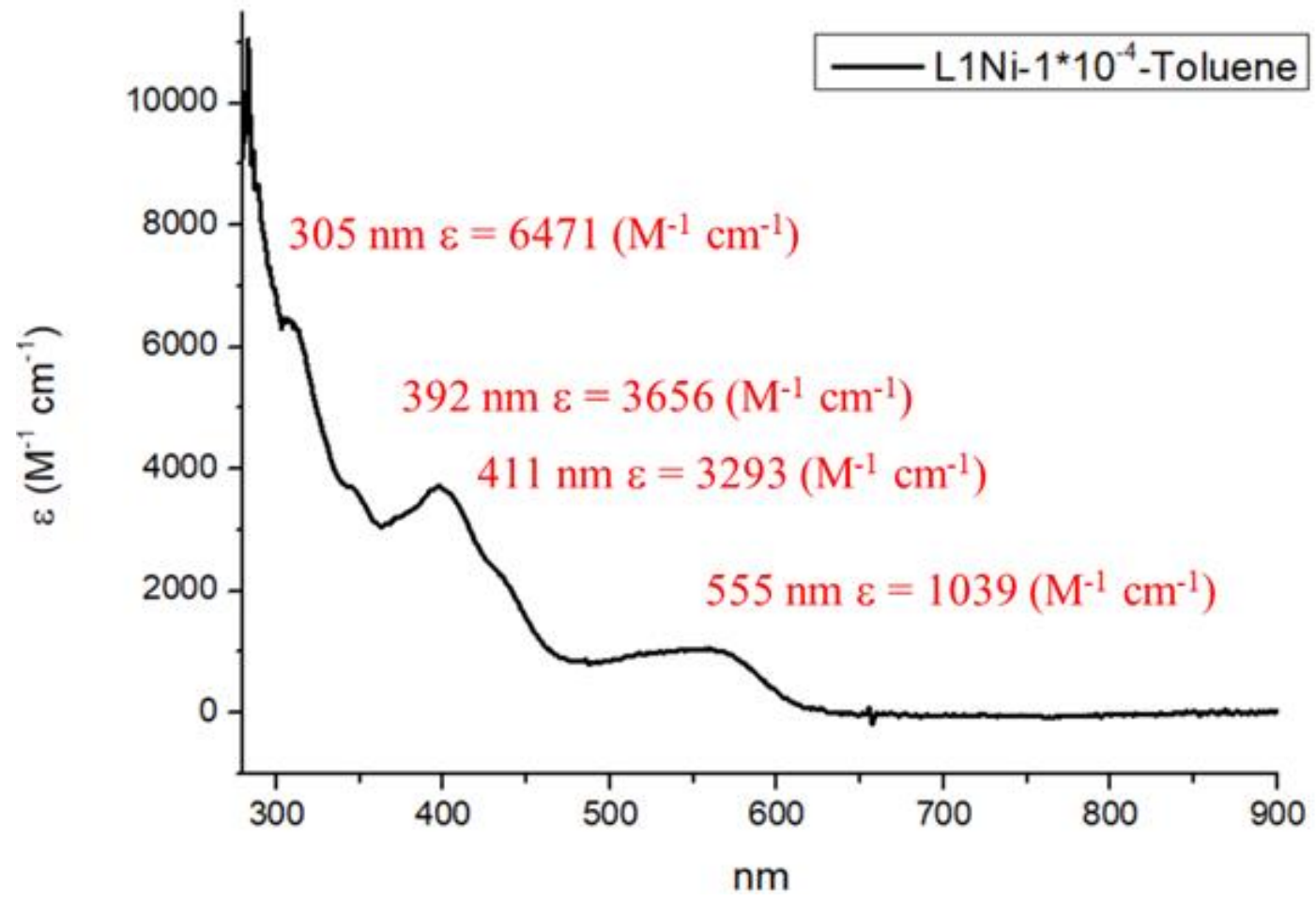

Figure S15. UV-vis absorption spectrum of **L1**Ni^{I} (toluene, 1 x 10^{-4} M). The complex of **L1**Ni^{I} was reported previously.[8]

L2Ni^{I} (Y-shaped) UV-vis (toluene, 298 K, ε in $M^{-1}cm^{-1}$): 311 (11999; charge transfer), 389 (2830; charge transfer), 406 (2514; d-d transition), 448 (1295; d-d transition), 569 (1637; d-d transition) nm.

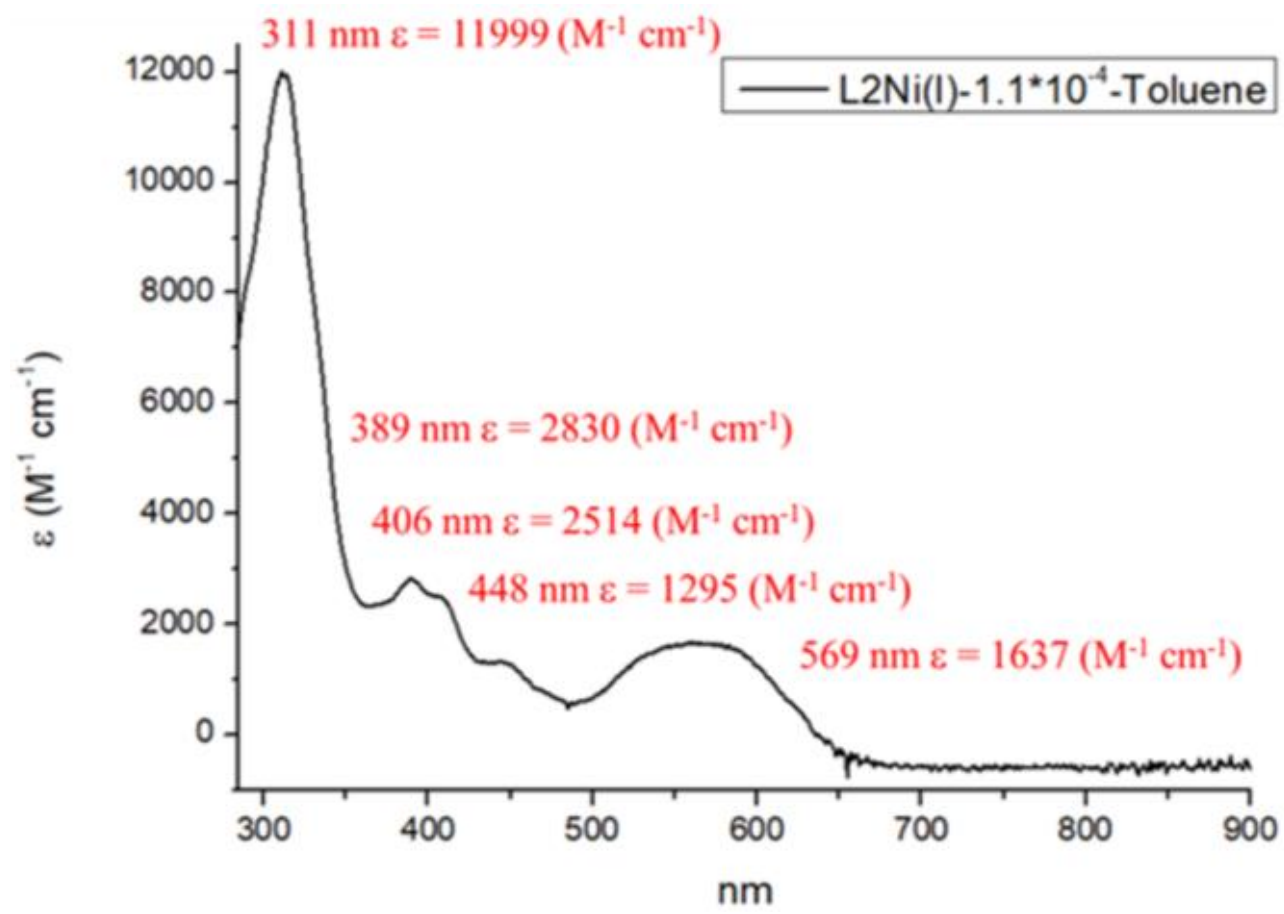

Figure S16. UV-vis absorption spectrum of **L2**Ni^{I} (toluene, 1.1 x 10^{-4} M).

[L2^{MeCNH}NiCl][Cl] (five-coordinate, high-spin Ni(II))

UV-vis (MeCN, 298 K, ε in $M^{-1}cm^{-1}$): 256 (8137; charge transfer), 335 (681; d-d transition), 626 (25; d-d transition) nm.

The strong UV band is LMCT. The weak, longer-wavelength d-d band at 626 nm (ε = 25) is typical of high-spin five-coordinate Ni(II) centers (distorted trigonal-bipyramidal, $\tau_5 \approx 0.60$). The intermediate band at 335 nm has moderate intensity consistent with the lower symmetry. This spectrum matches the magnetic moment and the crystallographically established ionic formulation arising from nitrile-induced backbone activation, a reactivity absent in the shorter-arm **L1** analogue.

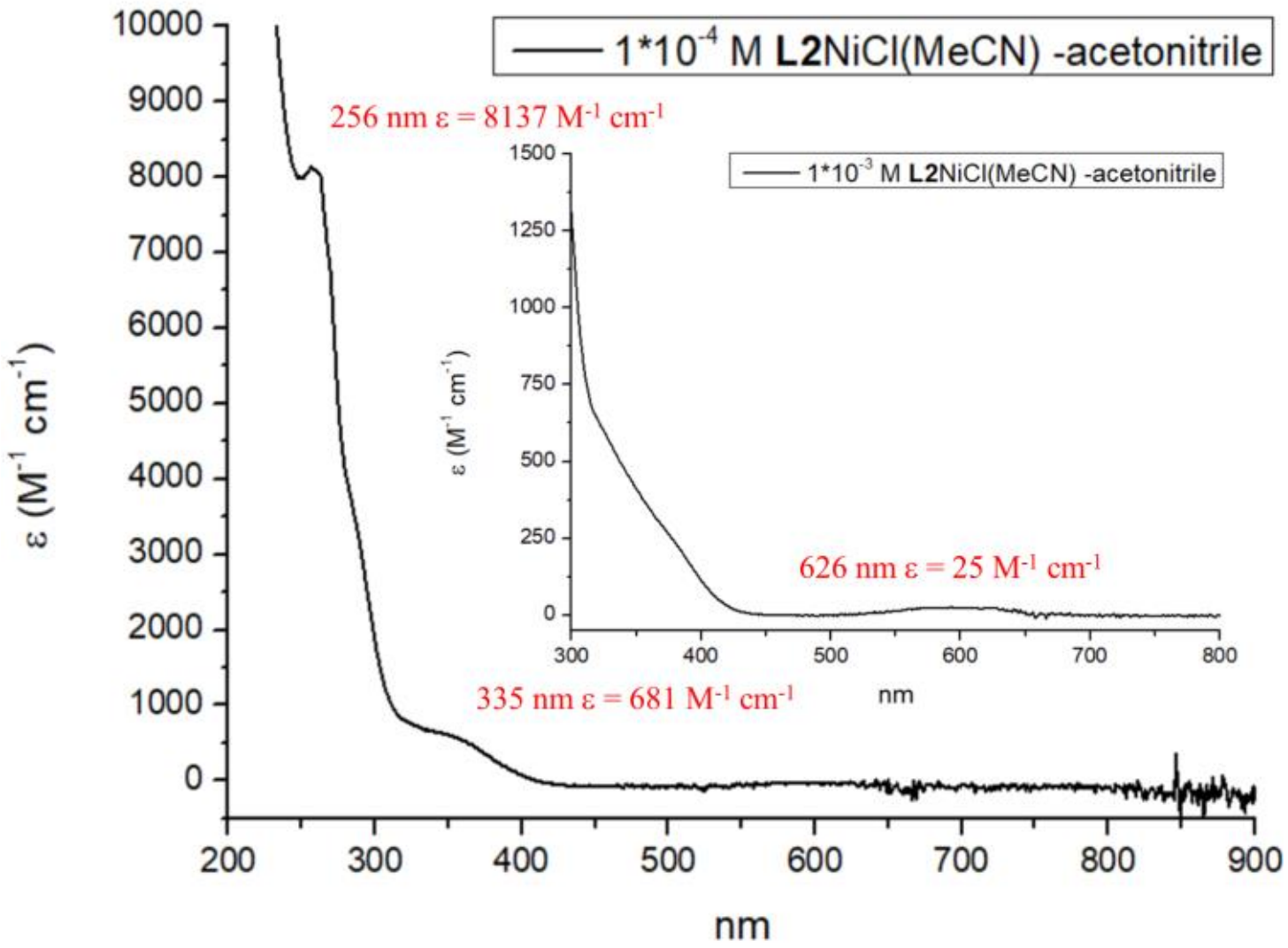

Figure S17. UV-vis absorption spectrum of [**L2**^{MeCNH}NiCl][Cl] (acetonitrile, 10^{-4} M).

L1NiOH (mononuclear square-planar terminal hydroxide)

UV-vis (toluene, 298 K, ε in $M^{-1}cm^{-1}$) 329 (4556; charge transfer), 342 (4195; charge transfer), 400 (3635; d-d transition), 430 (sh, 2199; d-d transition), 579 (98; d-d transition) nm.

The intense UV features are LMCT. The very weak 579 nm band is diagnostic of square-planar low-spin Ni(II). The higher-ε bands near 400-430 nm likely contain significant charge-transfer character mixed with d-d transitions (common in β-diketiminate systems); pure d-d transitions rarely exceed a few hundred in ε. The spectrum is fully consistent with the crystallographically confirmed mononuclear terminal-OH structure and the diamagnetic ^{1}H NMR (including the characteristic upfield Ni-OH resonance).

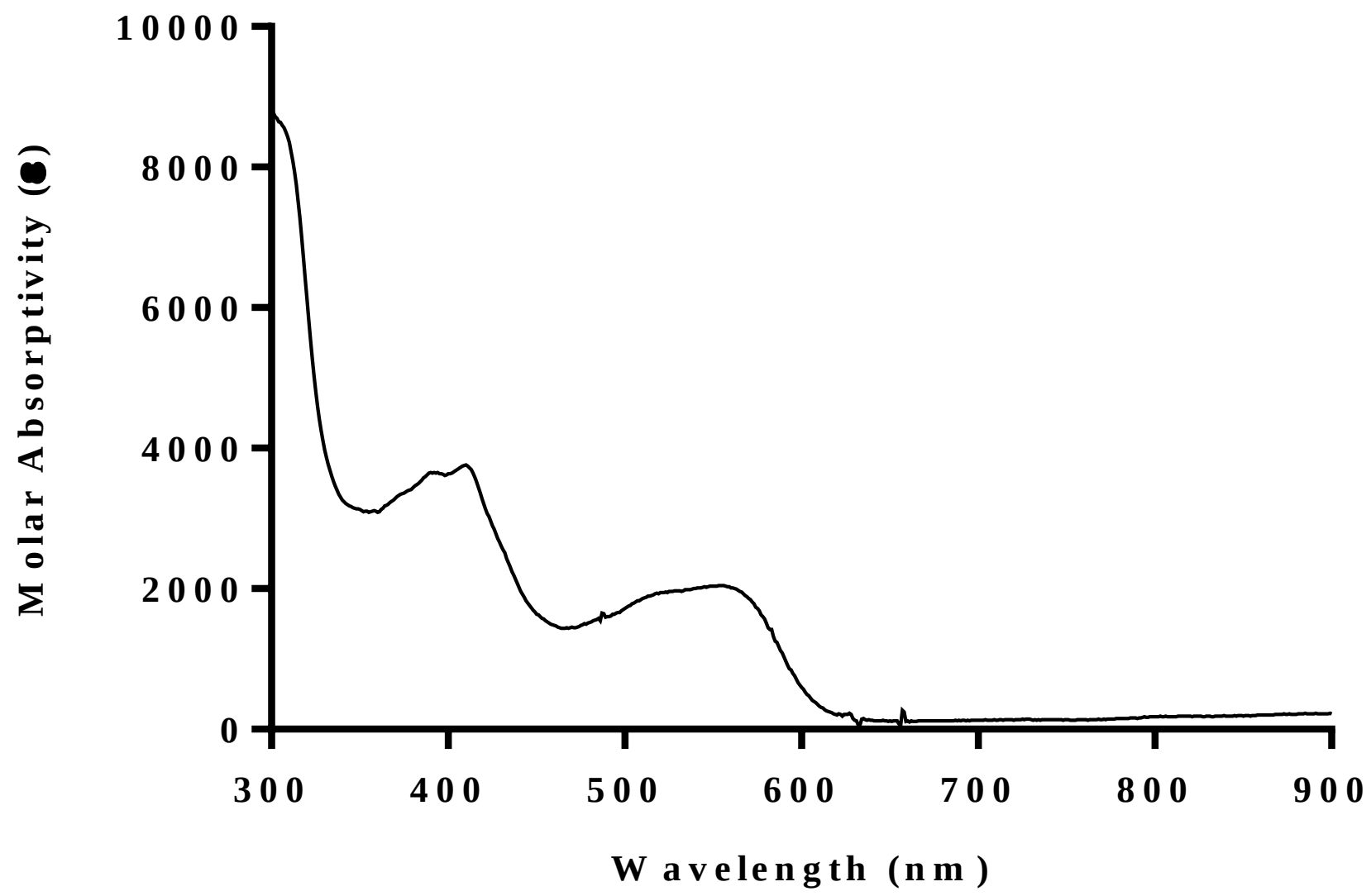

Figure S18. UV-vis absorption spectrum of **L1**NiOH (toluene, 10^{-4} M). The complex of **L1**NiOH was reported previously.[8]

L2Ni(μ-OH)$_2$Ni**L2** (dinuclear square-planar bridging hydroxides)

UV-vis (toluene, 298 K, ε in $M^{-1}cm^{-1}$): 321 (6020; charge transfer), 382 (3990; charge transfer), 454 (sh, 475; d-d transition), 609 (47; d-d transition) nm.

Strong LMCT bands again dominate the UV region. The weak visible d-d absorptions match those expected for two independent square-planar low-spin Ni(II) centers. The pendant pyridine arms remain non-coordinated, illustrating how the longer ethyl linker permits dimerization with bridging hydroxides while preserving the preferred square-planar geometry at each metal.

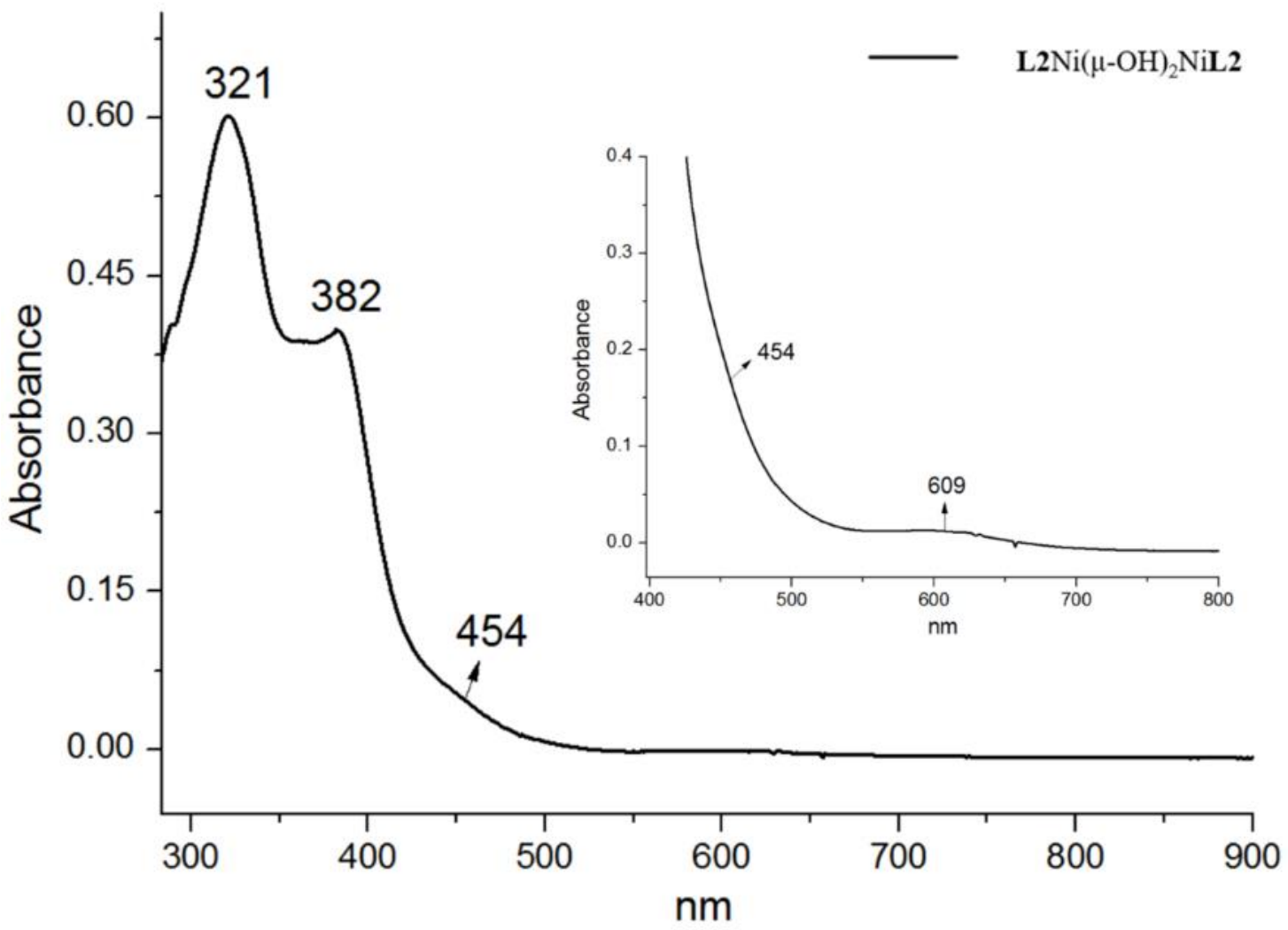

Figure S19. UV-vis absorption spectrum of **L2**Ni(μ-OH)$_2$Ni**L2** (toluene, 10^{-4} M).

Summary of the UV-vis spectroscopy characterization.

The UV-vis data provide independent spectroscopic corroboration of the crystallographic and magnetic results. Extension of the pyridyl arm from methylene (**L1**) to ethylene (**L2**) systematically distorts the Ni(II) chloride from square-planar (weak d-d) to seesaw (stronger d-d + paramagnetism). These intensity and energy patterns align with established trends for Ni(II)/Ni(I) complexes: low-symmetry or high-spin species exhibit more intense d-d bands, while square-planar low-spin d^8 centers show the classic weak visible absorptions. The spectra therefore reinforce the central thesis that chelate-ring size is a powerful handle for tuning geometry, spin multiplicity, and reactivity in this unsymmetrical β-diketiminate platform.

SI. Mass Spectrometry

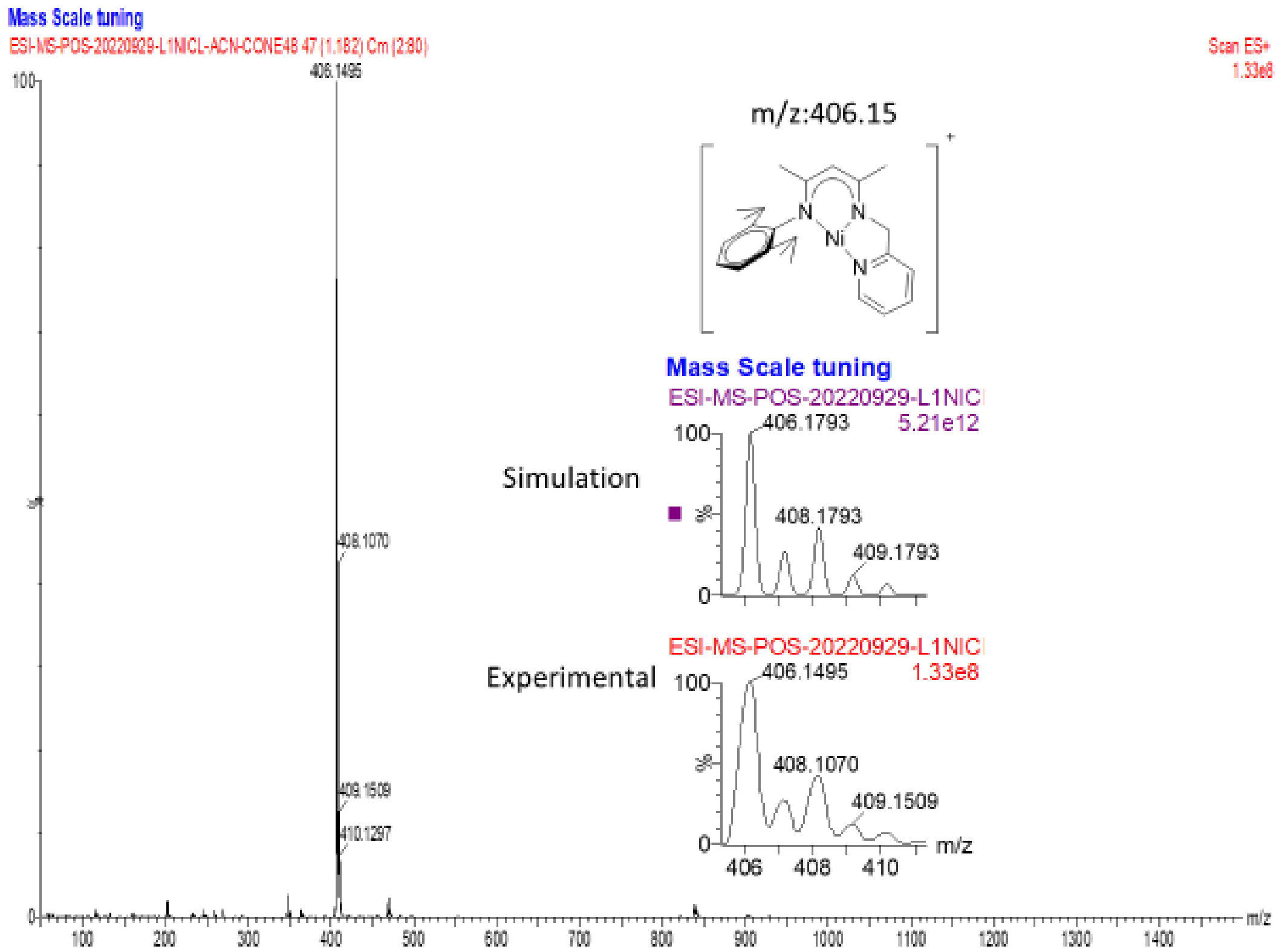

Figure S20. ESI-MS spectrum of **L1**Ni^{II}Cl (in acetonitrile). The complex of **L1**Ni^{II}Cl was reported previously.[8]

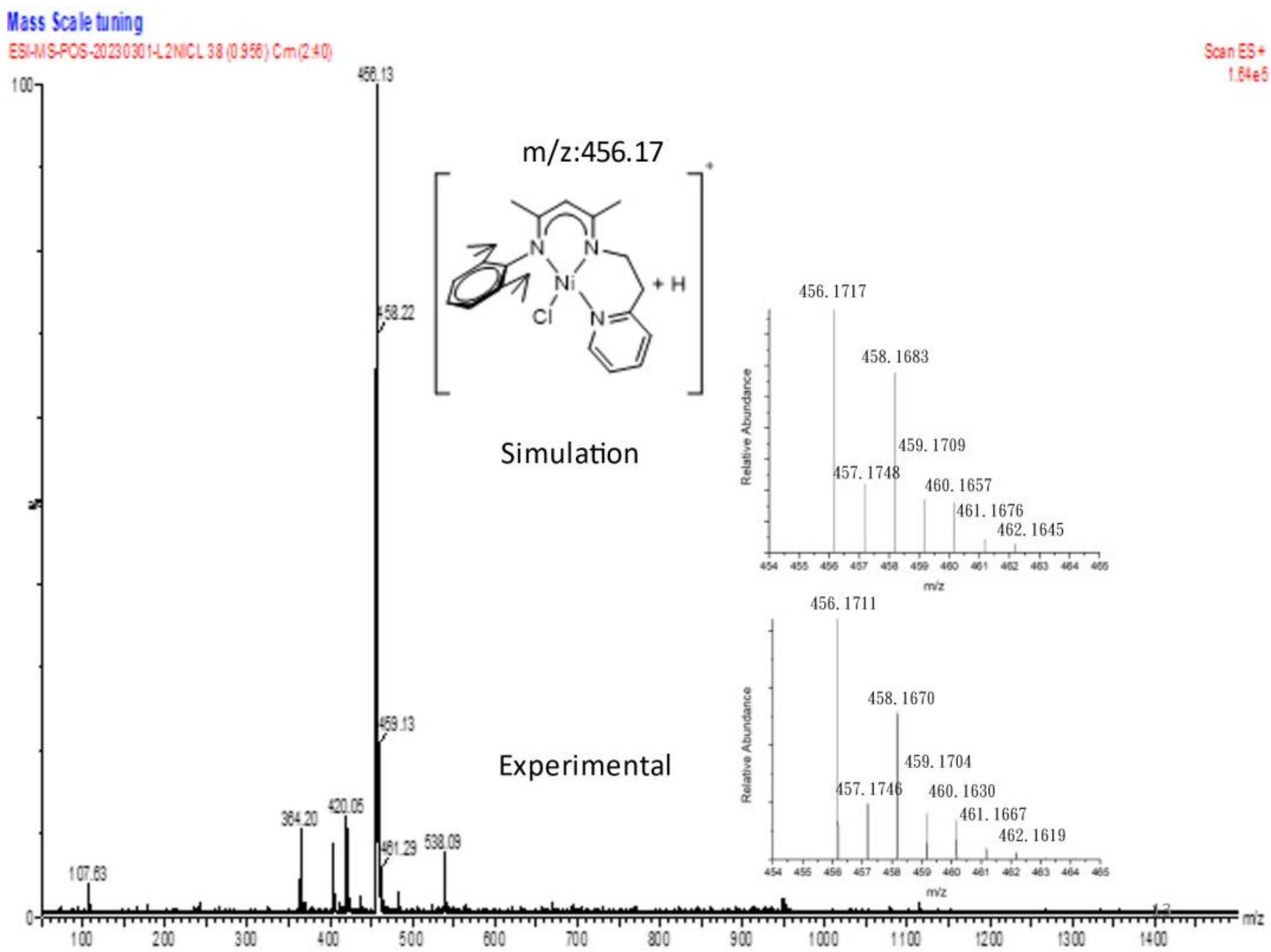

Figure S21. HR-MS spectrum of **L2**Ni^{II}Cl (in THF).

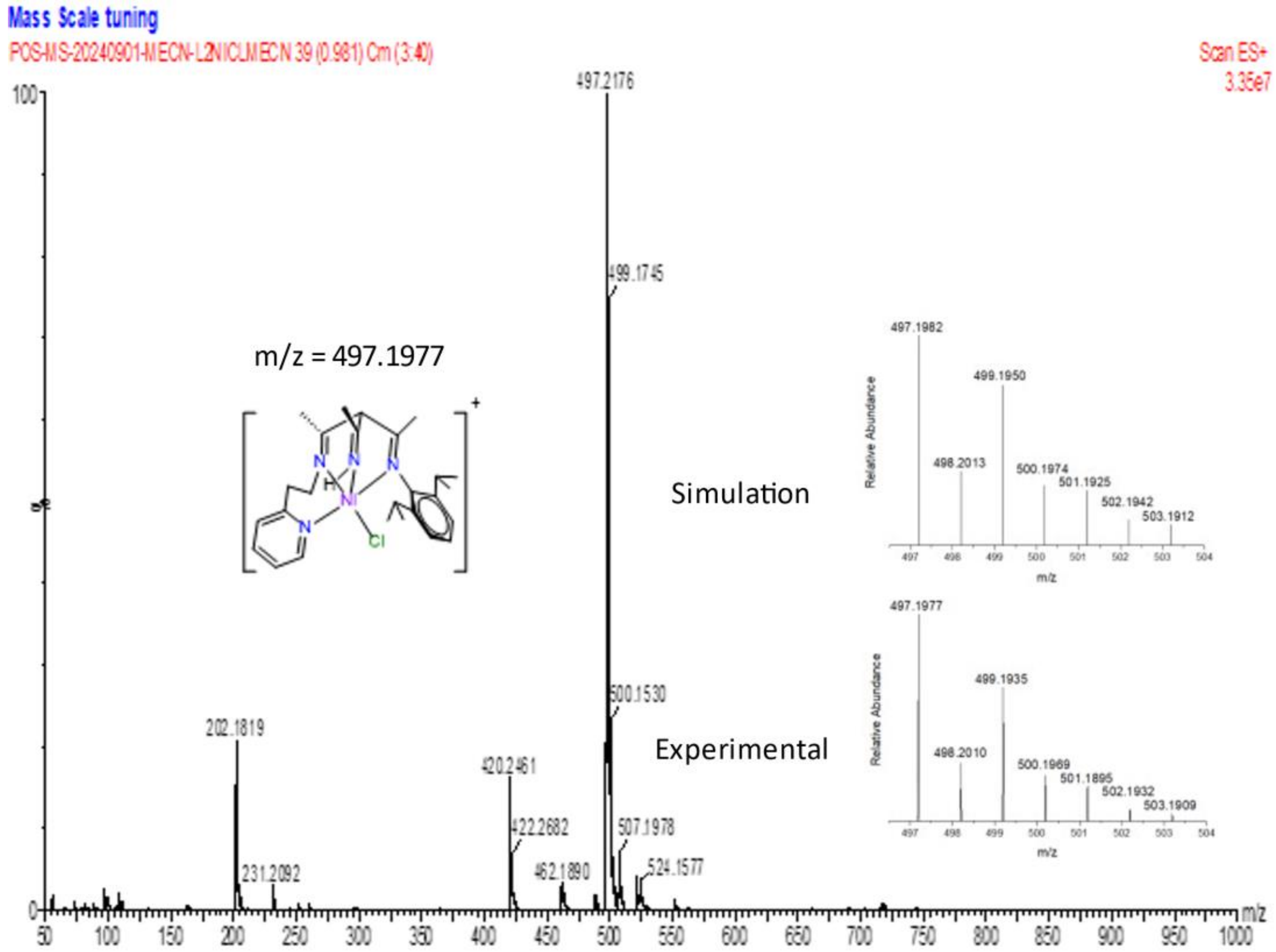

Figure S22. HR-MS spectrum of [**L2**^{MeCNH}NiCl]$^+$ (in acetonitrile).

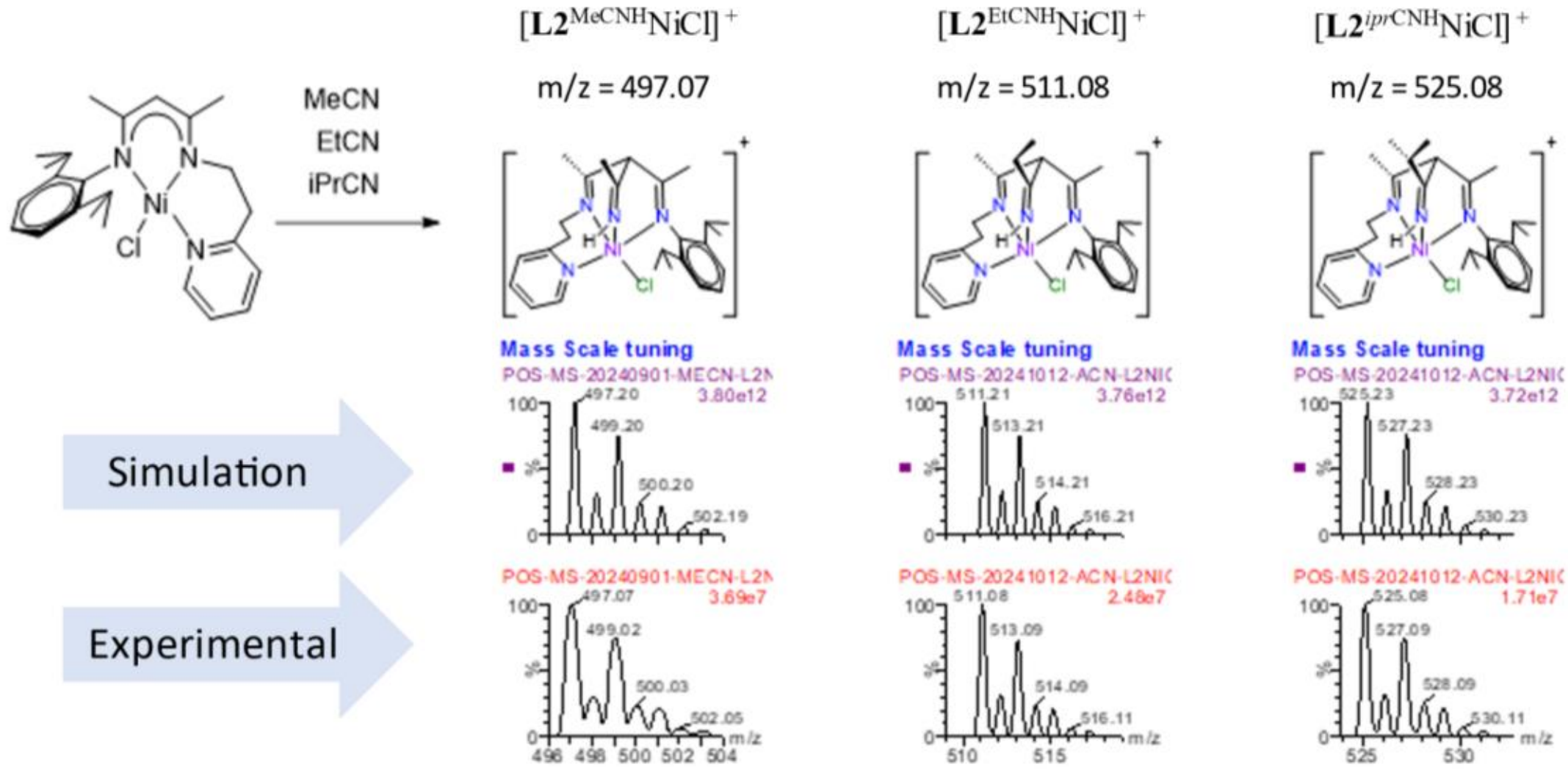

Figure S23. ESI-MS spectrum of [**L2**^{MeCNH}NiCl]$^+$, [**L2**^{EtCNH}NiCl]$^+$ and [**L2**$^{ipr\mathrm{CNH}}$NiCl]$^+$ (in acetonitrile).

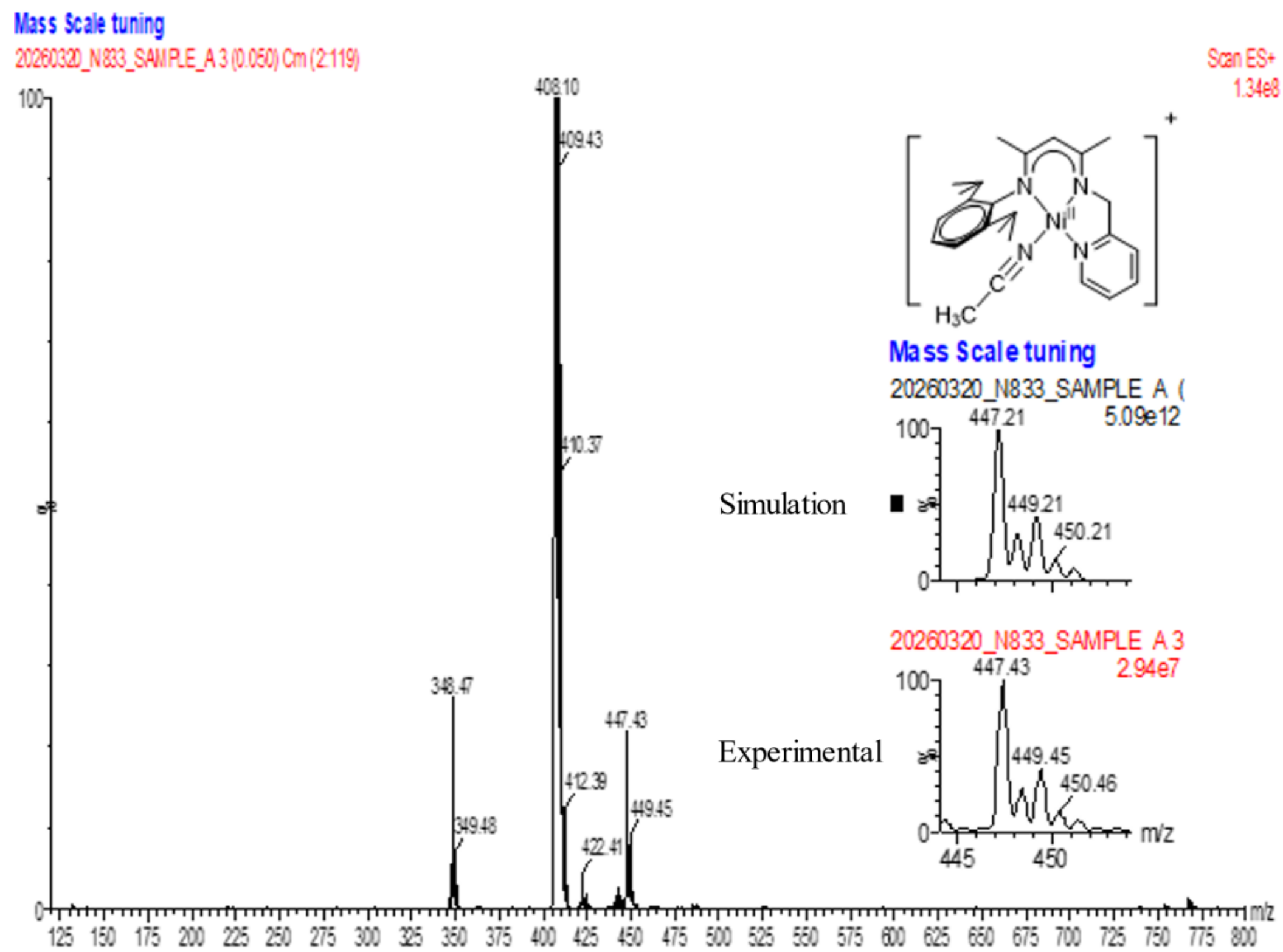

Figure S24. ESI-MS spectrum of **L1**$Ni^{II}Cl$ in acetonitrile in the presence of HCl/Et_2O (in acetonitrile).

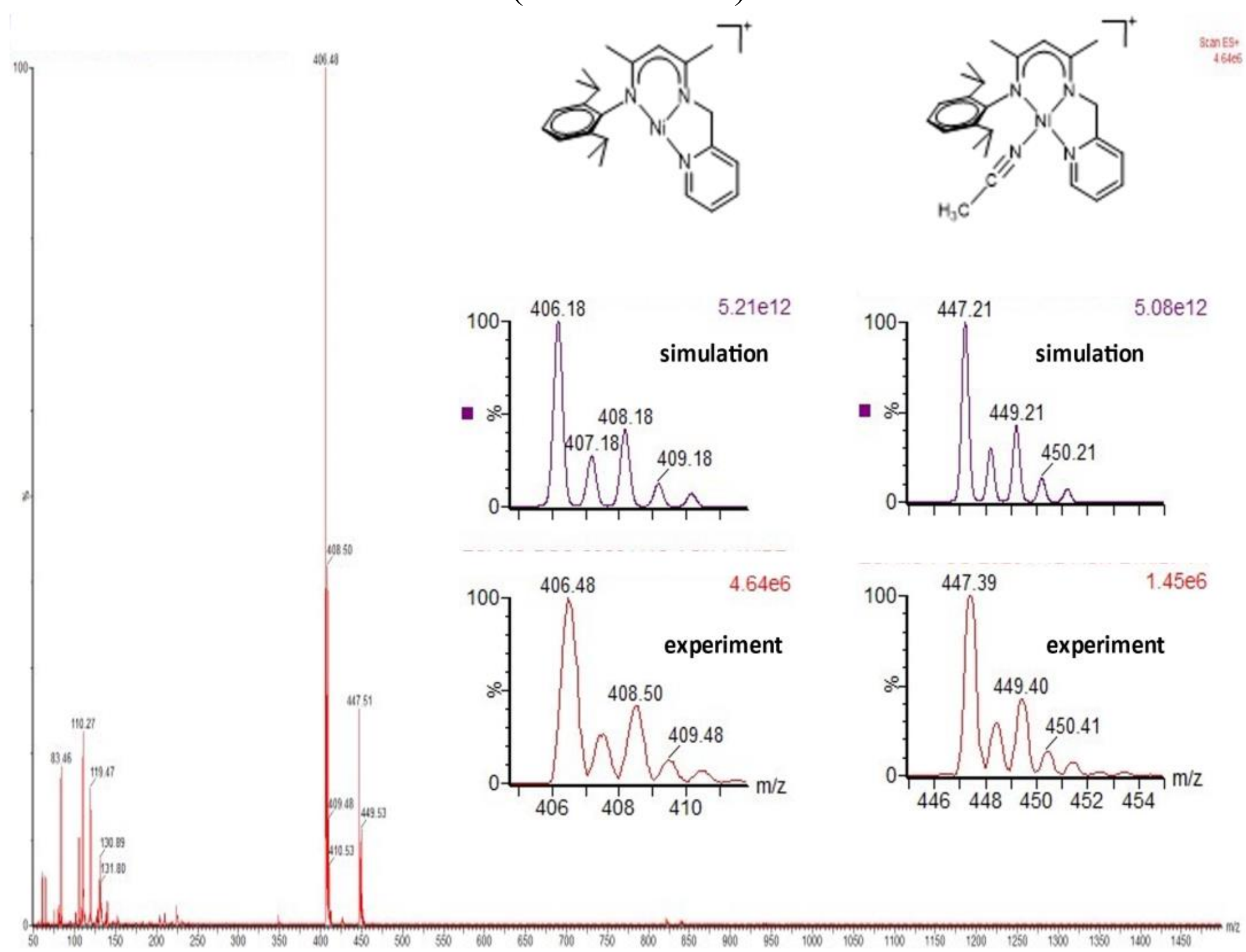

Figure S25. ESI-MS spectrum of independently synthesized [**L1**Ni(MeCN)]$^+$[CF_3SO_3]$^-$ in acetonitrile.

SI. FT-IR Spectra

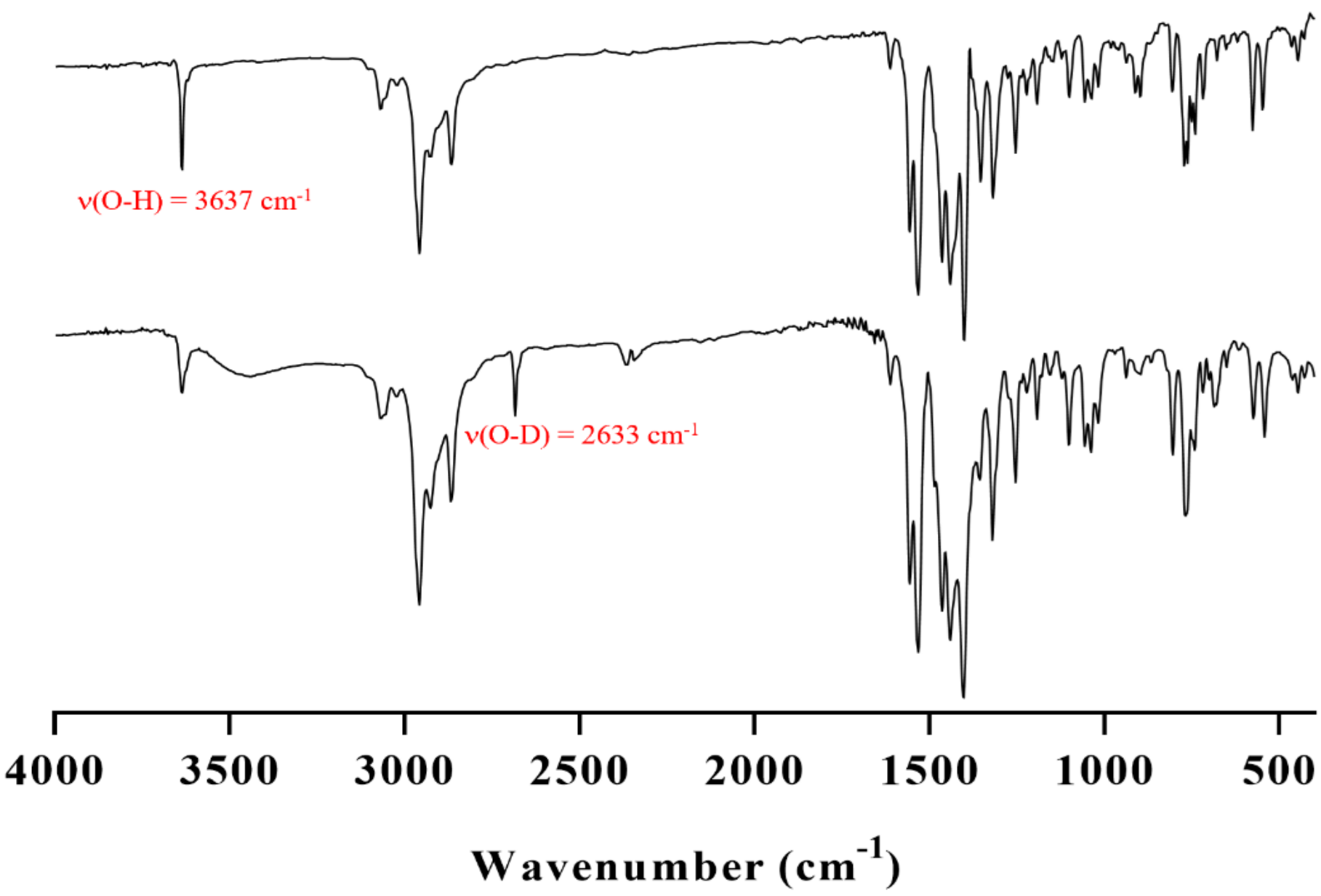

Figure S26. FT-IR spectrum (KBr) of **L1**NiOH (top) and its deuteration product **L1**NiOD (bottom). The complex of **L1**NiOH was reported previously.[8]

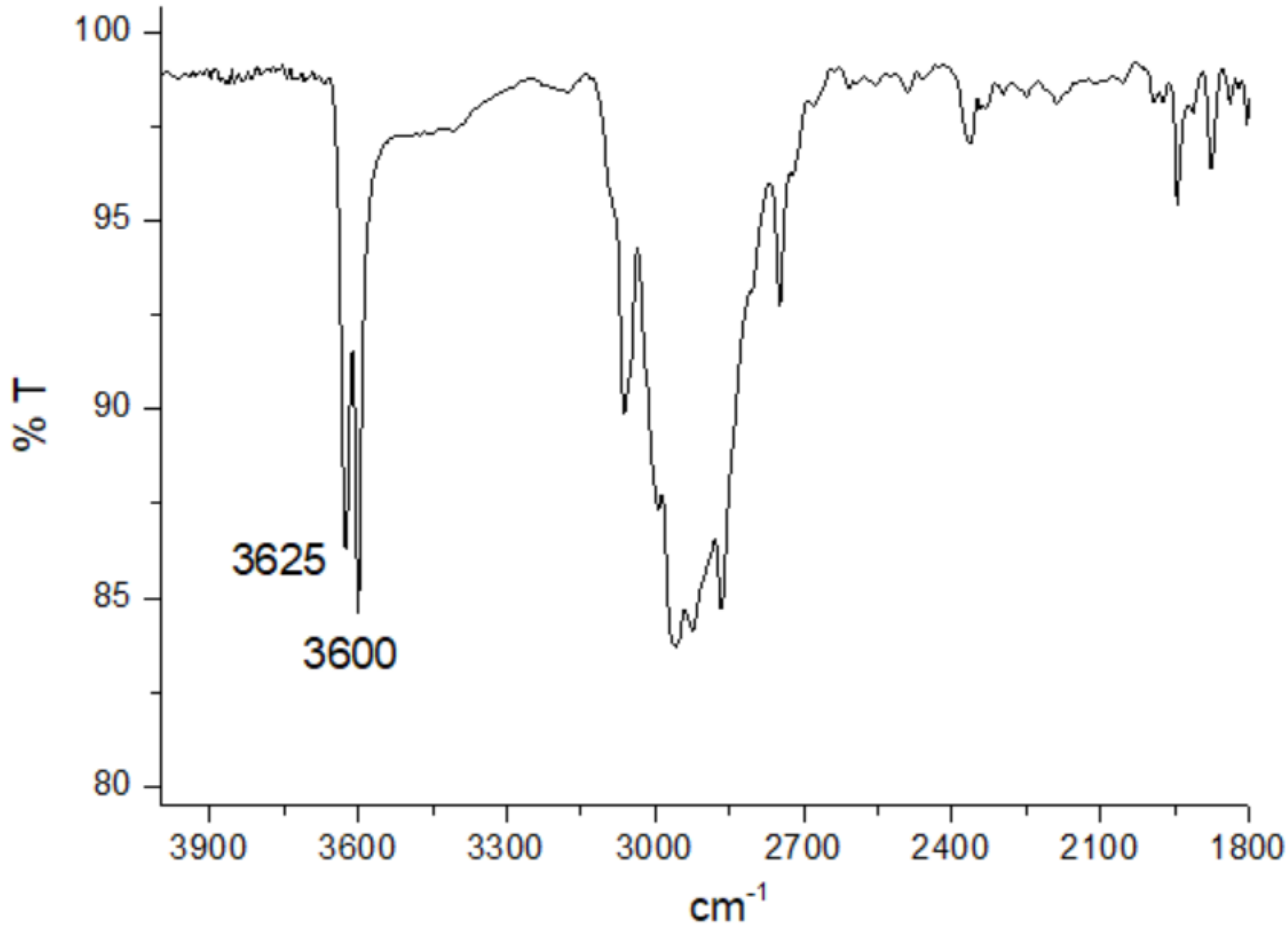

Figure S27. FT-IR spectrum (KBr) of **L2**Ni(μ-OH)$_2$Ni**L2**.

SI. Crystal Data

Table S1. Selected bond lengths (Å) and angles (degrees) for **L2**$Ni^{II}Cl$ and **L2**Ni^{I}.

	**L2**$Ni^{II}Cl$	**L2**Ni^{I}
Ni(1)-N(1)	1.8948(18)	1.8665(15)
Ni(1)-N(2)	1.8780(19)	1.9210(15)
Ni(1)-N(3)	1.9113(19)	1.9083(15)
Ni(1)-Cl(1)	2.2137(6)	
C-C	1.391(3)	1.397(3)
	1.400(3)	1.407(3)
C-N	1.335(3)	1.332(2)
	1.318(3)	1.331(2)
N(1)-Ni(1)-N(2)	94.24(8)	98.63(6)
N(2)-Ni(1)-N(3)	90.41(8)	101.23(7)
N(1)-Ni(1)-N(3)	159.91(8)	159.37(6)
N(2)-Ni(1)-Cl(1)	157.96(6)	
∠NCCCN, aryl[a]	86.41	81.89

[a.] ∠angle between the NCCCN backbone plane and *N*-aryl ring.

Table S2. Crystallographic data for complexes **L2**$Ni^{II}Cl$ and **L2**Ni^{I}.

	**L2**$Ni^{II}Cl$	**L2**Ni^{I}
Empirical formula	$C_{24}H_{32}ClN_3Ni$	$C_{24}H_{32}N_3Ni$
Formula weight	456.68	421.23
T (K)	130(2)	130(2)
Crystal size (mm^3)	0.50 × 0.30 × 0.20	0.40 × 0.20 × 0.20
Crystal system	Orthorhombic	Monoclinic
Space group	Pbca	$P2_1/c$
a (Å)	10.0846(2)	16.6525(4)
b (Å)	16.6354(3)	9.3125(2)
c (Å)	27.6478(4)	15.3860(4)
α (deg)	90	90
β (deg)	90	110.521(3)
γ (deg)	90	90
V ($Å^3$)	4638.23(14)	2234.60(10)
Z	8	4
D_{calcd} (g cm^{-3})	1.308	1.252
μ (mm^{-1})	0.966	0.882
Reflections measured	51841	29194
Independent reflections	4074	3928
Data/restraints/parameters	4074 / 144 / 268	3928 / 0 / 259
GOF on F^2	1.041	1.061
Rint	0.0402	0.0341
R_1 [$I > 2\sigma(I)$]	0.0335	0.0293
R_1 (all data)	0.0375	0.0366
wR_2 [$I > 2\sigma(I)$]	0.0865	0.0718
wR_2 (all data)	0.0887	0.0744
Largest diff. peak/hole (e^-/ $Å^3$)	0.949 / −0.389	0.432 / −0.229

Table S3. Selected bond lengths (Å) and angles (degrees) for [**L1**Ni(MeCN)][CF_3SO_3] and [**L2**^{MeCNH}NiCl][Cl].

	[**L1**Ni(MeCN)][CF_3SO_3]	[**L2**^{MeCNH}NiCl][Cl]
Ni(1)-N(1)	1.8899(19)	2.119(6)
Ni(1)-N(2)	1.8489(19)	2.033(6)
Ni(1)-N(3)	1.927(2)	2.089(6)
Ni(1)-N(4)$_{(MeCN)}$	1.897(2)	2.004(6)
Ni(1)-Cl(1)		2.256(2)
	1.383(3)	1.543(10)
C-C	1.391(3)	1.510(10)
		1.518(10)
	1.340(3)	1.282(9)
C-N	1.327(3)	1.255(9)
		1.255(9)
N(1)-Ni(1)-N(2)	94.05(8)	86.2(3)
N(2)-Ni(1)-N(3)	85.03(8)	87.4(2)
N(1)-Ni(1)-N(3)	176.67(8)	173.6(3)
N(2)-Ni(1)-N(4)	174.06(8)	89.6(2)
N(2)-Ni(1)-Cl(1)		137.38(18)
N(4)-Ni(1)-Cl(1)		132.99(18)
∠NCCCN, aryl[a]	89.39	51.54

[a] ∠angle between the NCCCN backbone plane and *N*-aryl ring.

Table S4. Crystallographic data for complexes [**L1**Ni(MeCN)][CF_3SO_3] and [**L2**^{MeCNH}NiCl][Cl].

	[**L1**Ni(MeCN)][CF_3SO_3]	[**L2**^{MeCNH}NiCl][Cl]
Empirical formula	$C_{26}H_{33}F_3N_4NiO_3S$	$C_{28}H_{39}Cl_2N_5Ni$
Formula weight	597.33	575.25
T (K)	100.15	200(2)
Crystal size (mm^3)	0.15 × 0.12 × 0.07	0.48 × 0.19 × 0.01
Crystal system	Monoclinic	Orthorhombic
Space group	$P2_1/n$	Pbca
a (Å)	13.8042(3)	19.8525(10)
b (Å)	13.4297(3)	13.9711(9)
c (Å)	15.6340(3)	23.3628(14)
α (deg)	90	90
β (deg)	106.428(2)	90
γ (deg)	90	90
V (Å^3)	2780.01(11)	6479.9(7)
Z	4	8
D_{calcd} (g cm^{-3})	1.427	1.179
μ (mm^{-1})	2.185	0.786
Reflections measured	44663	106749
Independent reflections	5513	5721
Data/restraints/parameters	5513 / 0 / 350	5721 / 79 / 307
GOF on F^2	1.054	1.086
Rint	0.0462	0.0896
R_1 [$I > 2\sigma(I)$]	0.0519	0.1090
R_1 (all data)	0.0538	0.1211
wR_2 [$I > 2\sigma(I)$]	0.1416	0.2306
wR_2 (all data)	0.1433	0.2375
Largest diff. peak/hole (e^-/ Å^3)	1.850 / −1.118	1.352 / −1.583

Table S5. Selected bond lengths (Å) and angles (degrees) for [H**L2**H][Cl] and **L2**Ni(μ-OH)$_2$Ni**L2**.

	[H**L2**H][Cl]		**L2**Ni(μ-OH)$_2$Ni**L2**	
	Molecule A	Molecule B	Molecule A	Molecule B
Ni(1)-N(1)			1.8696(16)	1.8710(16)
Ni(1)-N(2)			1.8808(17)	1.8846(17)
Ni(1)-O(1)			1.8696(16)	1.8813(19)
$Ni^{i}(1)$-$O^{i}(1)$			1.8716(17)	1.8746(19)
C-C	1.405(7)	1.390(7)	1.375(3)	1.382(3)
	1.399(6)	1.382(7)	1.394(3)	1.397(3)
C-N	1.316(6)	1.331(6)	1.333(3)	1.334(2)
	1.320(6)	1.335(6)	1.329(3)	1.319(3)
N(1)-Ni(1)-O(1)			169.11(7)	167.72(9)
N(2)-Ni(1)-$O^{i}(1)$			172.07(7)	167.58(8)
N(1)-Ni(1)-N(2)			95.39(7)	95.06(7)
∠NCCCN, aryl[a]	82.26	82.17	76.67	78.44

[a.] ∠angle between the NCCCN backbone plane and *N*-aryl ring.

Table S6. Crystallographic data for complexes [H**L2**H][Cl] and **L2**Ni(μ-OH)$_2$Ni**L2**.

	[H**L2**H][Cl]	**L2**Ni(μ-OH)$_2$Ni**L2**[a]
Empirical formula	$C_{48}H_{68}Cl_2N_6$	$C_{48}H_{66}N_6Ni_2O_2$
Formula weight	799.98	876.48
T (K)	99.99(13)	130(2)
Crystal size (mm^3)	0.09 × 0.07 × 0.03	0.40 × 0.30 × 0.20
Crystal system	Orthorhombic	Monoclinic
Space group	$Pna2_1$	I2/a
a (Å)	20.3871(4)	22.9898(7)
b (Å)	8.2962(2)	17.2624(6)
c (Å)	27.6957(6)	24.7849(7)
α (deg)	90	90
β (deg)	90	105.538(3)
γ (deg)	90	90
V (Å^3)	4684.32(18)	9476.6(5)
Z	4	8
D_{calcd} (g cm^{-3})	1.134	1.229
μ (mm^{-1})	1.527	0.837
Reflections measured	38018	54676
Independent reflections	8065	9938
Data/restraints/parameters	8065 / 1 / 518	9938 / 1 / 543
GOF on F^2	1.009	1.039
Rint	0.0493	0.0513
R_1 [$I > 2\sigma(I)$]	0.0609	0.0419
R_1 (all data)	0.0725	0.0602
wR_2 [$I > 2\sigma(I)$]	0.1510	0.1073
wR_2 (all data)	0.1607	0.1174
Largest diff. peak/hole (e$^-$/ Å^3)	1.007 / −0.253	0.440 / −0.274

[a] Regarding Alert B in the checkCIF report of **L2**Ni(μ-OH)$_2$Ni**L2**, there is no suitable acceptor close to the terminal OH hydrogen atoms. Explanation: The presence of the terminal OH groups was unambiguously confirmed by solid-state FT-IR.

SI. References

(1) Marenich, A. V.; Cramer, C. J.; Truhlar, D. G. Universal Solvation Model Based on Solute Electron Density and on a Continuum Model of the Solvent Defined by the Bulk Dielectric Constant and Atomic Surface Tensions. *J. Phys. Chem. B* **2009**, *113* (18), 6378-6396. DOI: https://doi.org/10.1021/jp810292n.

(2) Wertz, D. H. Relationship between the gas-phase entropies of molecules and their entropies of solvation in water and 1-octanol. *J. Am. Chem. Soc.* **1980**, *102* (16), 5316-5322. DOI: https://doi.org/10.1021/ja00536a033.

(3) Abraham, M. H. Relationship between solution entropies and gas phase entropies of nonelectrolytes. *J. Am. Chem. Soc.* **1981**, *103* (22), 6742-6744. DOI: https://doi.org/10.1021/ja00412a036.

(4) Chen, H.-Y.; Lin, Y.-F. DFT study on the catalytic decomposition of hydrogen peroxide by iron complexes of nitrilotriacetate. *J. Comput. Chem.* **2023**, *44* (26), 2058-2072. DOI: https://doi.org/10.1002/jcc.27179.

(5) Chen, H.-Y.; Lin, Y.-F. Cobalt(II) Aqua Complex-Mediated Hydrogen Peroxide Activation: Possible Roles of HOOOH and Co(II)-OOOH Intermediates in Singlet Oxygen Generation. *Inorg. Chem.* **2025**, *64* (1), 554-562. DOI: https://doi.org/10.1021/acs.inorgchem.4c03966.

(6) Chen, H.-Y.; Lin, Y.-F. Cobalt(II)-Mediated Fenton-like Reactions: Effects of Second-Sphere H_2O_2 and Thiolate Coordination. *Inorg. Chem.* **2026**, *65* (1), 557-567. DOI: https://doi.org/10.1021/acs.inorgchem.5c04687.

(7) Frisch, M. J.; Trucks, G. W.; Schlegel, H. B.; Scuseria, G. E.; Robb, M. A.; Cheeseman, J. R.; Scalmani, G.; Barone, V.; Petersson, G. A.; Nakatsuji, H.; et al. Gaussian 16. Revision C.02; Gaussian, Inc. *Wallingford, CT* **2016**.

(8) Chen, S.-H.; Yang, T.-H.; Chang, Y.-L.; Lin, W.-S.; Chuang, C.-H.; Yang, C.-H.; Chen, H.-Y.; Tsai, M.-L.; Hsu, S. C. N. Identifying a Terminal Nickel-Oxygen Complex Bearing an Unsymmetrical β-Diketiminate Ligand. *JACS Au* **2026**, *6* (5), 2709-2714. DOI: https://doi.org/10.1021/jacsau.6c00356.
